# The Anti-Repressor MecR2 Promotes the Proteolysis of the *mecA* Repressor and Enables Optimal Expression of β-lactam Resistance in MRSA

**DOI:** 10.1371/journal.ppat.1002816

**Published:** 2012-07-26

**Authors:** Pedro Arêde, Catarina Milheiriço, Hermínia de Lencastre, Duarte C. Oliveira

**Affiliations:** 1 CREM, Department of Life Sciences, Faculdade de Ciências e Tecnologia, Universidade Nova de Lisboa, Caparica, Portugal; 2 Laboratory of Molecular Genetics, Instituto de Tecnologia Química e Biológica, Universidade Nova de Lisboa, Oeiras, Portugal; 3 Laboratory of Microbiology and Infectious Diseases, The Rockefeller University, New York, New York, United States of America; University of Tubingen, Germany

## Abstract

Methicillin-resistant *Staphylococcus aureus* (MRSA) is an important human pathogen, which is cross-resistant to virtually all β-lactam antibiotics. MRSA strains are defined by the presence of *mecA* gene. The transcription of *mecA* can be regulated by a sensor-inducer (MecR1) and a repressor (MecI), involving a unique series of proteolytic steps. The induction of *mecA* by MecR1 has been described as very inefficient and, as such, it is believed that optimal expression of β-lactam resistance by MRSA requires a non-functional MecR1-MecI system. However, in a recent study, no correlation was found between the presence of functional MecR1-MecI and the level of β-lactam resistance in a representative collection of epidemic MRSA strains. Here, we demonstrate that the *mecA* regulatory locus consists, in fact, of an unusual three-component arrangement containing, in addition to *mecR1-mecI*, the up to now unrecognized *mecR2* gene coding for an anti-repressor. The MecR2 function is essential for the full induction of *mecA* expression, compensating for the inefficient induction of *mecA* by MecR1 and enabling optimal expression of β-lactam resistance in MRSA strains with functional *mecR1-mecI* regulatory genes. Our data shows that MecR2 interacts directly with MecI, destabilizing its binding to the *mecA* promoter, which results in the repressor inactivation by proteolytic cleavage, presumably mediated by native cytoplasmatic proteases. These observations point to a revision of the current model for the transcriptional control of *mecA* and open new avenues for the design of alternative therapeutic strategies for the treatment of MRSA infections. Moreover, these findings also provide important insights into the complex evolutionary pathways of antibiotic resistance and molecular mechanisms of transcriptional regulation in bacteria.

## Author Summary

Methicillin-resistance *Staphylococcus aureus* (MRSA) is an important human pathogen, causing a wide range of infections. MRSA strains are resistant to virtually all β-lactam antibiotics and often are also resistant to many other classes of antibiotics, leaving physicians with few therapeutic options. MRSA is defined by the presence of the *mecA* gene. The induction of *mecA* transcription in response to β-lactams involves a unique series of proteolytic steps and some critical details of this signal transduction mechanism are still illusive. For instance, it is not fully explained why the induction of *mecA* by its cognate regulatory genes *mecR1-mecI* appears to be very inefficient and it is not clear if the observed MecI repressor proteolysis is mediated directly by the activated MecR1 sensor-inducer. In this study, we demonstrate that the *mecA* regulatory locus is not a two-component system but instead it is a three-component system containing the previously unrecognized anti-repressor *mecR2* gene. MecR2 disturbs the binding of the repressor MecI to the *mecA* promoter, which leads to its proteolytic inactivation independently from MecR1. Moreover, our data shows that in the presence of functional *mecR1-mecI* genes, *mecR2* is essential for a robust induction of *mecA* transcription and, as consequence, for the optimal expression of β-lactam resistance.

## Introduction

Methicillin-resistant *Staphylococcus aureus* (MRSA) is a leading cause of infections in hospitals in many countries and has also become an important community- and livestock-associated pathogen [1]–[3]. Recently, a report from CDC has reassessed the burden of MRSA infections in the USA, putting the number of deaths attributable to MRSA in front of those related to HIV-AIDS, Alzheimer disease or homicide [4]. MRSA are resistant to virtually all β-lactam antibiotics, one of the most clinically relevant class of antimicrobial agents. In addition, contemporary MRSA strains are frequently resistant to many other antimicrobial classes leaving clinicians with few therapeutic options.

The MRSA characteristic phenotype is due to an extra penicillin-binding protein (PBP2a) coded by the *mecA* gene [5], which has a remarkable reduced affinity for many β-lactams [6]. In addition, >95% of MRSA strains have also a β-lactamase enzyme coded by *blaZ* that confers penicillin-resistance [7]–[9]. The *mecA* transcription can be controlled by the divergent *mecR1-mecI* regulatory genes, coding for a sensor-inducer and a repressor, respectively [10]. This genetic organization of the *mecA* locus is similar to that of the β-lactamase, which contains the structural gene *blaZ* and the homologous *blaR1-blaI* regulatory genes. In fact, there is a cross-talk between both systems [11]–[14], and the signal-transduction mechanisms are believed to be identical [15], [16], involving two proteolytic steps, in contrast with the most common bacterial signal transduction mechanism that involves the phosphorylation of regulatory proteins [17]. Specifically, the currently accepted model of *mecA* regulation involves two main steps: (i) binding of the β-lactam antibiotic to the extracellular sensor domain of MecR1 leads to the autocatalytic cleavage of the sensor-inducer and activation of the cytoplasmatic inducer domain, which appears to be a prometalloprotease; (ii) the activated inducer domain of MecR1 either directly cleaves the promoter-bound MecI dimers or promotes the repressor cleavage, which disables the ability of the repressor protein to dimerize and bind to the *mecA* promoter, enabling the expression of the resistance gene. MecR1 once cleaved can no longer transmit signal but, since the expression of *mecR1-mecI* is also up-regulated, the *mecA* induction continues as long as the antibiotic is present in the environment.

Some details of the signaling mechanism involved in the transcriptional control of *mecA* are still elusive. For instance, induction of *mecA* by MecR1 has been described as extremely slow [12], [13], so that cells with intact *mecR1-mecI* regulatory system appear phenotypically susceptible in spite of the presence of *mecA* – the so-called “pre-MRSA” phenotype [10], [18]. Based on these observations, it has been postulated that high-level resistance to β-lactams, characteristic of many contemporary MRSA clinical strains, implies a non-functional *mecR1-mecI* regulatory system. In agreement with this hypothesis, several studies have described the accumulation of point mutations and/or gene deletions in the *mecR1-mecI* coding sequences [19]–[23]. Still, in some of these studies [19], [21], [23], based on contradictory observations, the existence of alternative *mecA* regulatory mechanisms has also been proposed. In fact, in a recent study, we could not establish any correlation between the *mecR1-mecI* integrity and the β-lactam resistance phenotype in a representative collection of epidemic MRSA strains and, unexpectedly, overexpression *in trans* of a wild-type copy of MecI had no effect on the phenotypic expression of resistance in most strains [24].

Here, we identify the missing link that explains the puzzling observations described above. We demonstrate that the *mecA* regulatory locus is in fact a three-component system that contains, besides *mecR1-mecI* genes, the *mecR2* gene, which is co-transcribed with *mecR1-mecI*. *In vitro* and *in vivo* assays show that MecR2 acts as an anti-repressor by interacting directly with MecI repressor, disturbing its binding to the *mecA* promoter, which results in its inactivation by proteolytic cleavage. In MRSA strains with functional *mecR1-mecI* genes, MecR2 is essential for the full induction of *mecA* transcription, compensating for the inefficient induction of *mecA* by *mecR1* and enabling the optimal expression of β-lactam resistance. These findings suggest a need to revise the current model for the induction of *mecA* expression in clinical MRSA strains and open new avenues for the design of alternative therapeutic strategies targeting the regulatory pathway of *mecA* expression. In addition, this unusual combination of repressor, sensor-inducer and anti-repressor, together with the unique modulation of a series of proteolytic cleavage steps underlying the signal transduction mechanism, provides important insights into the evolution of antibiotic-resistance and transcriptional control of genes in bacteria.

## Results/Discussion

### The *mecA* cognate regulatory locus is a three-component system

**Figure 1:**
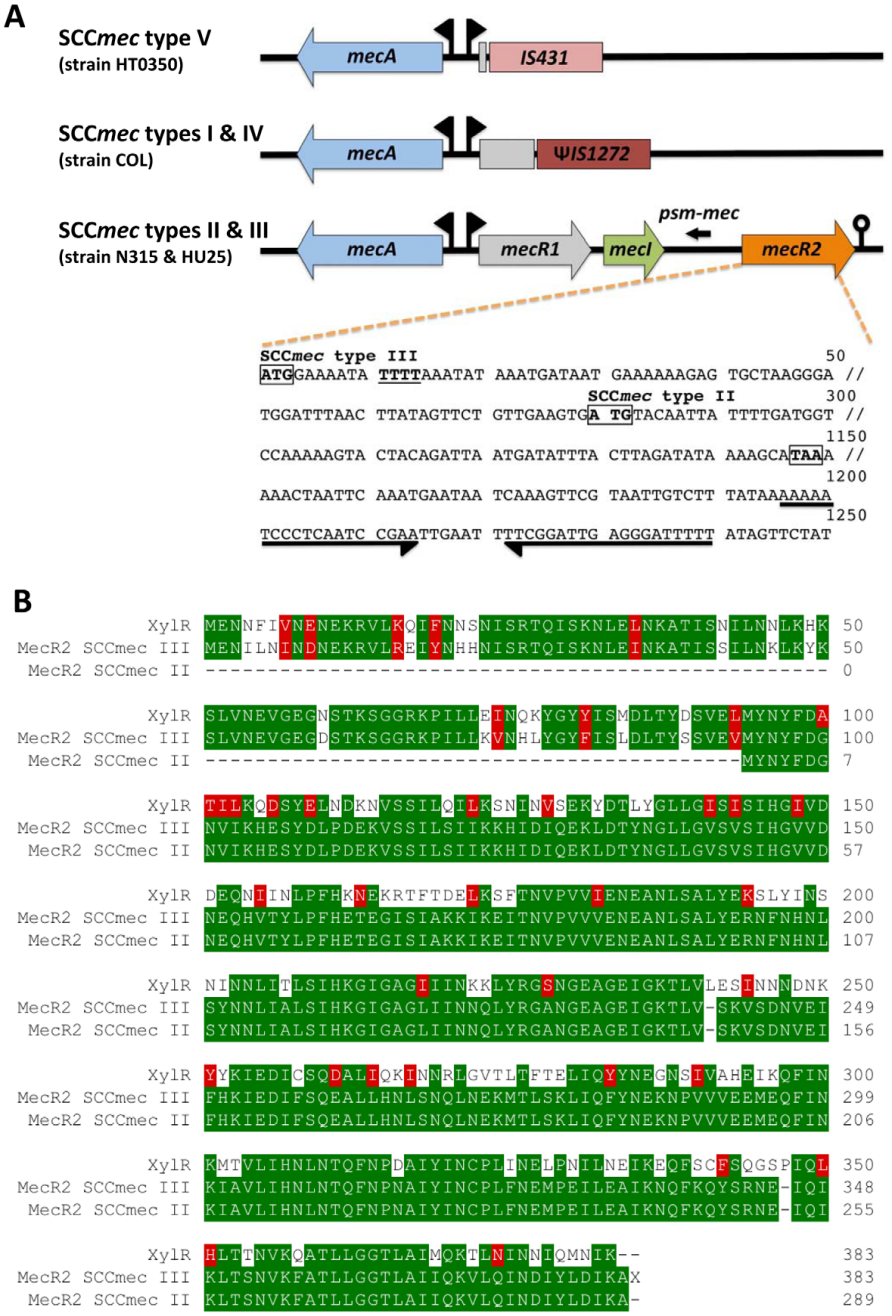
The *mecA* regulatory locus is a three-component system. (A) Genetic organization of the *mecA* regulatory locus in major SCC*mec* types I–V and prototype MRSA strains used in this study. The magnified DNA sequence shows the two *mecR2* start codons in SCC*mec* type III and III (boxed), the stop codon (boxed), the four-thymine deletion in SCC*mec* type II (underlined), and the putative terminator (inverted arrows). (B) Multiple sequence alignment between the repressor of the xylose operon (XylR) and MecR2 from prototype SCC*mec* types II and III strains. Green – identical residues; red – similar residues; white – divergent residues. The figure was prepared using “The Sequence Manipulation Suite” freely available at http://www.bioinformatics.org.

Since the MRSA phenotype is not affected by the overexpression *in trans* of the *mecA* repressor [24], we hypothesized that a third regulator might be involved in the *mecA* transcriptional control. Taking into account that *mecA* gene is part of a large polymorphic exogenous DNA fragment (the so called SCC*mec* element), which has integrated in the chromosome [25], we reasoned that the putative additional regulator should be located within this chromosomal cassette, most likely upstream to the *mecA* gene; i.e. genetically linked to the *mecR1-mecI* region. Upon analysis of prototype sequences of SCC*mec* types II and III, which are characterized by complete *mecR1-mecI* coding sequences [26], we found a highly conserved region (99.9% homology) immediately downstream of *mecI*. This region contains the divergent small coding sequence for a phenol-soluble modulin, *psm*-*mec*, involved in *S. aureus* virulence and colony spreading [27], [28], and a putative open-reading frame (ORF) that, due to a difference in four-tandem thymine residues, has a variable length (Figure 1A): 870 bp in SCC*mec* type II prototype strain N315 (accession no. D86934, positions 41794-40925) and 1149 bp in SCC*mec* type III prototype strain HU25 (accession no. AF422694, positions 4729-3861). Both variants are homologous to the repressor of the xylose operon of *S. xylosus*, XylR (accession no. X57599) with an amino-acid identity of 60–64%; the four-thymine deletion in strain N315 eliminates the N-terminal DNA-binding helix-turn-helix domain of XylR (Figure 1B). Available genomic and SCC*mec* sequence data demonstrate that both ORF variants are disseminated in *S. aureus* and in other staphylococcal species containing SCC*mec* sequences (Figure S1). We coined the name of *mecR2* for this putative ORF.

Analysis of the upstream sequences of the putative *mecR2* gene revealed no obvious promoter sequences. A putative terminator region, consisting of two perfect inverted repeats of 19 base pairs, was identified downstream the stop codon of *mecR2*. No terminator sequences were found in the *mecI-mecR2* intergenic region, suggesting that the putative *mecR2* gene might be co-transcribed together with *mecR1-mecI* from the *mecR1* promoter. This hypothesis was confirmed by transcriptional analysis of *mecR2* by reverse-transcriptase PCR (RT-PCR) in prototype strains N315 and HU25. Using internal primers for the putative coding region of *mecR2* a positive signal was detected in both prototype strains. Moreover, using pairs of primers spanning the *mecI-mecR2* and *mecR1*-*mecI* regions, positive signals were detected suggesting that *mecR1-mecI-mecR2* genes are co-transcribed from the *mecR1* promoter (Figure S2).

### *mecR2* is involved in the optimal expression of β-lactam resistance

**Figure 2:**
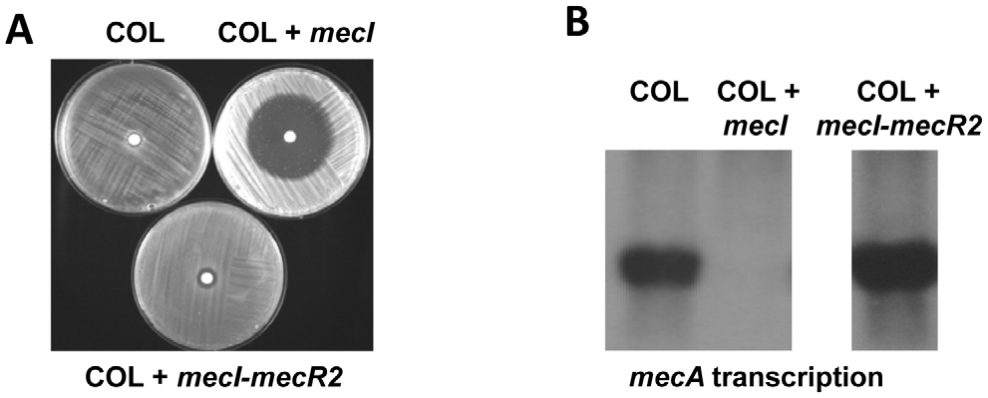
*mecR2* interferes with the *mecI*-mediated repression of β-lactam resistance. (A) Co-overexpression of *mecI* and *mecR2* region (COL+*mecI-mecR2*) reverted the effect of *mecI* overexpression (COL+*mecI*) on the oxacillin-resistance phenotype in strain COL, as evaluated with diffusion disks containing 1 mg of oxacillin. (B) Northern blotting analysis of *mecA* transcription shows that in the presence of *mecR2* locus the repressor effect of *mecI* is reverted.

We next evaluated the role of *mecR2* on the MRSA phenotype by constructing a series of recombinant strains using two parental strains with contrasting phenotypes. The first of these, strain COL is highly resistant to methicillin, has no *mecI*, has a partially deleted *mecR1*, expresses *mecA* constitutively, is negative for β-lactamase [29], and is *mecR2* negative. The second, strain N315 has a low-level methicillin-resistance phenotype, carries wild-type *mecR1-mecI* sequences, has an inducible expression of *mecA*, carries a β-lactamase plasmid [10], [18], and is *mecR2* positive. In previous studies, we have observed a sharp decrease in resistance to oxacillin in strain COL overexpressing *in trans* the repressor *mecI* (COL+*mecI*), whereas the great majority of other MRSA strains tested, including strain N315, did not show alterations in the oxacillin-resistance phenotype [24]; being oxacillin a methicillin analogue that has replaced methicillin in clinical use. In this study, we have cloned the *mecI-mecR2* region from strain N315 in the same plasmid vector. When strain COL was transformed with this recombinant plasmid (COL+*mecI-mecR2*) the resistant-phenotype was completely restored and so was the constitutive expression of *mecA* (Figure 2).

**Figure 3:**
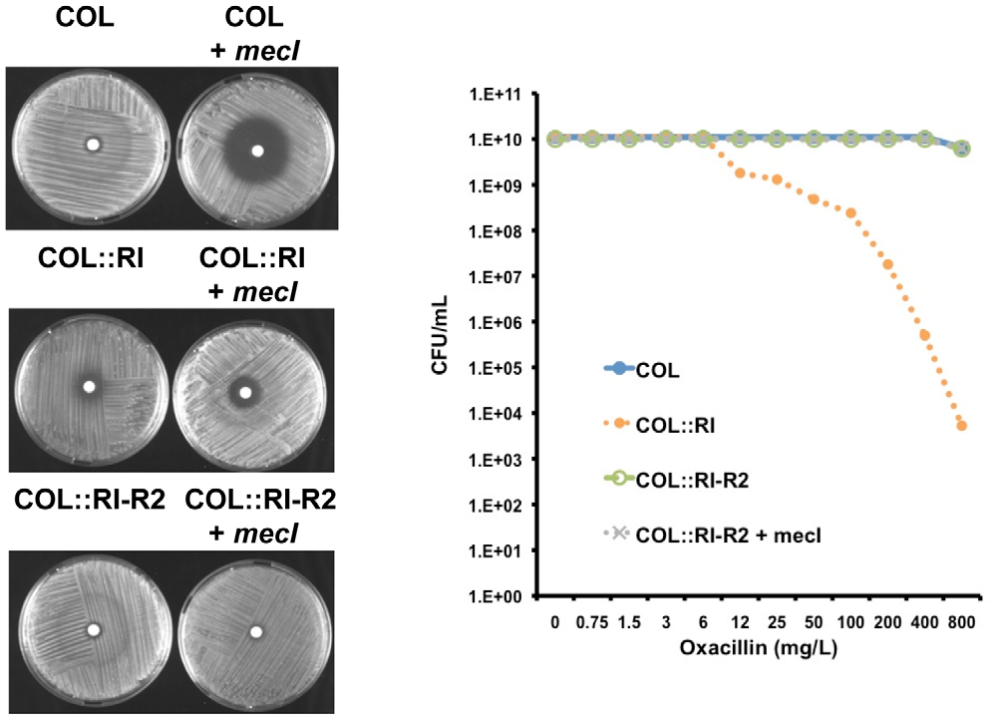
Reconstruction of the *mecA* regulatory locus in prototype strain COL. Reconstruction of the *mecR1-mecI* locus in the chromosome of strain COL (COL::RI) causes a decrease of the resistance level to oxacillin, which can be reverted by the reconstruction of the full *mecA* regulatory locus, *mecR1-mecI-mecR2* (COL::RI-R2). Control experiments with *mecI* overexpressed in trans (COL::RI+*mecI* and COL::RI-R2+*mecI*) demonstrate that the functions of *mecR1* and *mecR2* are not affected by high levels of MecI. For comparative purposes the overexpression of *mecI* in parental strain COL (COL+*mecI*) is also shown. The oxacillin-resistance levels were evaluated by diffusion disks containing 1 mg of oxacillin (left) or by population analysis profiles (PAP's) (right).

To exclude possible artifacts due to the overexpression of genes from multi-copy plasmids, we reconstructed the *mecR1-mecI-mecR2* regulatory locus of prototype strain N315 in the chromosome of strain COL, using an insertion-deletion strategy with a thermosensible plasmid (Figure S3). First, we inserted the wild-type sequences of *mecR1-mecI* (strain COL::RI), which caused a decrease of oxacillin-resistance when compared to the parental strain COL, in agreement with the poor induction of *mecA* by MecR1 alone (Figure 3). Compared to COL+*mecI* (Figure 2), the decrease of oxacillin-resistance in COL::RI was less severe, most likely due to the presence of the inducer MecR1. Upon introduction of the complete *mecA* regulatory locus; i.e. *mecR1-mecI-mecR2* (strain COL::RI-R2), the phenotype of parental strain COL was fully restored. As control experiments, we re-introduced the recombinant plasmid over-expressing *mecI* in recombinant strains COL::RI and COL::RI-R2, originating strains COL::RI+*mecI* and COL::RI-R2+*mecI*, respectively. In both cases no effect was detected on the phenotypic expression of resistance, suggesting that the functions of *mecR1* and *mecR2* are not affected when *mecI* is overexpressed *in trans*, in line with our previously reported observations [24]. Altogether, these experiments suggest that *mecR2* compensates for the poor induction of *mecA* by *mecR1*, enabling the optimal expression of resistance.

**Figure 4:**
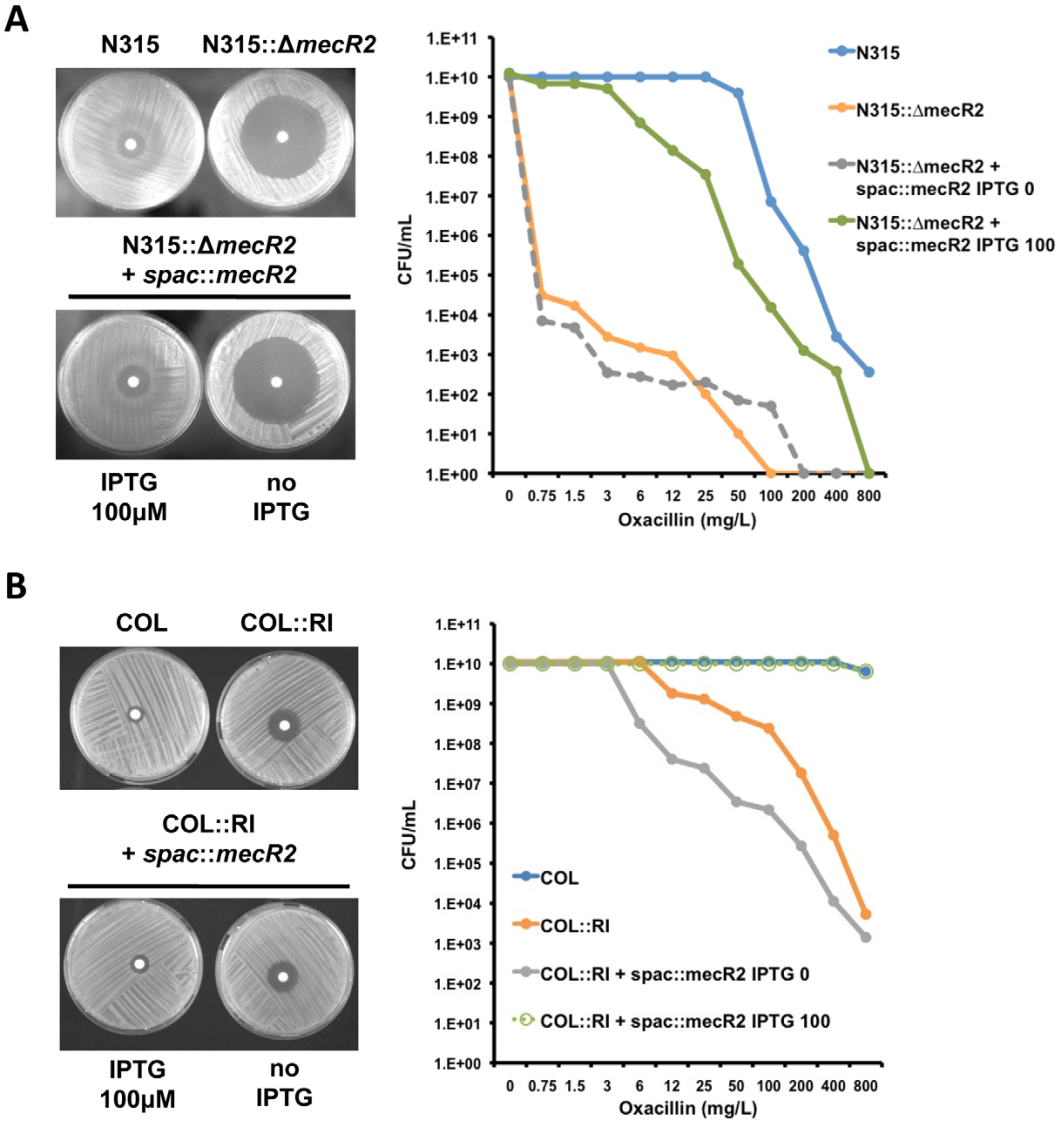
Role of *mecR2* on the optimal expression of β-lactam resistance. (A) Deletion of *mecR2* from the chromosome of strain N315 (N315::Δ*mecR2*) causes a decrease on the resistance level to oxacillin, which can be reverted upon complementation with *mecR2* expressed from an inducible promoter (N315::Δ*mecR2*+*spac*::*mecR2*) in the presence of the inducer (IPTG 100 µM). (B) The poor expression of oxacillin resistance by recombinant strain COL::RI, can also be reverted upon complementation with *mecR2* expressed from an inducible promoter (COL::RI+*spac*::*mecR2*) in the presence of the inducer (IPTG 100 µM). The oxacillin-resistance levels were evaluated by diffusion disks containing 1 mg of oxacillin (left) and by population analysis profiles (PAP's) (right).

To further clarify the *mecR2* function, the chromosomal copy of *mecR2* gene from strain N315 was replaced by an antibiotic-resistance marker (N315::Δ*mecR2*), using a similar insertion-deletion strategy (Figure S3). Deletion of *mecR2* caused a sharp decrease in the phenotypic expression of oxacillin-resistance in strain N315. We then cloned the *mecR2* gene from strain N315 under the control of an inducible promoter (*spac*::*mecR2*) and, in the presence of the inducer (IPTG 100 µM), we succeeded in complementing the *mecR2* null-mutant in strain N315 (Figure 4A) and restored the COL phenotype of recombinant strain COL::RI (Figure 4B). Since in these experiments only the *mecR2* gene was deleted from the chromosome of strain N315 and complemented *in trans*, it can be concluded that the intergenic *mecI-mecR2* region has no role in the phenotypic expression of β-lactam resistance. Altogether, these observations demonstrated that *mecR2* interferes with the *mecI*-mediated repression of *mecA*, compensates for the inefficient MecR1-mediated induction of *mecA* and enables the optimal expression of β-lactam resistance in the presence of functional *mecR1-mecI* sequences.

Of note, first attempts to complement the *mecR2* null-mutant by overexpression it *in trans* only succeeded if *mecR2* was co-overexpressed together with *mecI* (data not shown). This requirement for low MecR2 cellular amounts and/or equimolar cellular amounts of MecI and MecR2, suggests that at high cellular concentrations MecR2 function may be lost, either due to oligomerization or (non-specific) interference with essential cellular targets. A classical example of the requirement for equimolar ratios between interacting proteins is the *Escherichia coli* helicase DnaB/replication factor DnaC complex, in which the replication is inhibited when DnaC is in excess [30].

### *mecR2* is required for the full induction of *mecA* transcription

**Figure 5:**
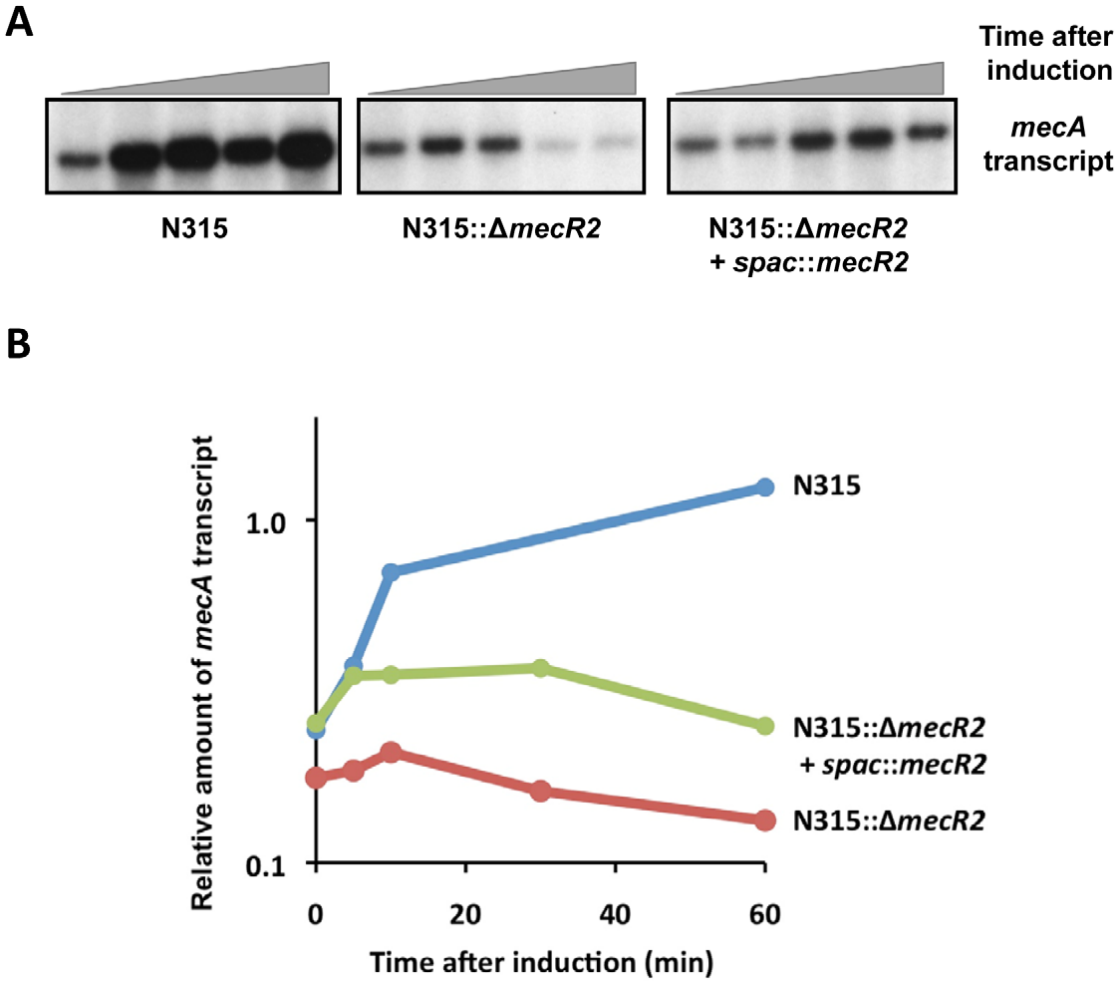
Effect of *mecR2* on the induction of *mecA* transcription. (A) Northern blot and (B) qRT-PCR analysis of the *mecA* induction profile in parental strain N315, *mecR2* null-mutant (N315::Δ*mecR2*) and complemented mutant (N315::Δ*mecR2*+*spac*::*mecR2*, IPTG 100 µM). Cultures were induced with a sub-MIC concentration of oxacillin (0.05 mg/L) and samples were taken at 0′, 5′, 10′ 30′ and 60′.

We next analyzed the effect of *mecR2* on the induction profile of *mecA* transcription in parental strain N315, its null-mutant for *mecR2*, and in the complemented mutant, by Northern blotting (Figure 5A) and quantitative Real-time RT-PCR (qRT-PCR) analysis (Figure 5B). In relative terms, upon induction with sub-MIC oxacillin, a much stronger induction of *mecA* transcription was observed in the parental strain than in the *mecR2* null-mutant (N315::Δ*mecR2*), in which the amount of *mecA* transcript seems to be not sustained during the last two time-points. In the complemented mutant (N315::Δ*mecR2*+spac::*mecR2*) there was a sustained induction of *mecA* transcription throughout the time-course of the experiment. However, in the complemented strain, although the resistant phenotype of the parental strain was fully restored (as illustrated in Figure 4A), the amount of *mecA* transcript was substantially lower and virtually identical to the *mecR2* null-mutant. Although this discordance is in agreement with previous studies reporting on the lack of a correlation between the cellular amounts of *mecA* transcript or protein and the phenotypic level of resistance [31], [32], we cannot formally exclude other possible explanations, such as MecR2 having multiple targets that affect the resistance phenotype. Nevertheless, these data suggest that the *mecR2* interferes with the induction of *mecA* transcription in response to β-lactams.

### *mecR2* transcription analysis

**Figure 6:**
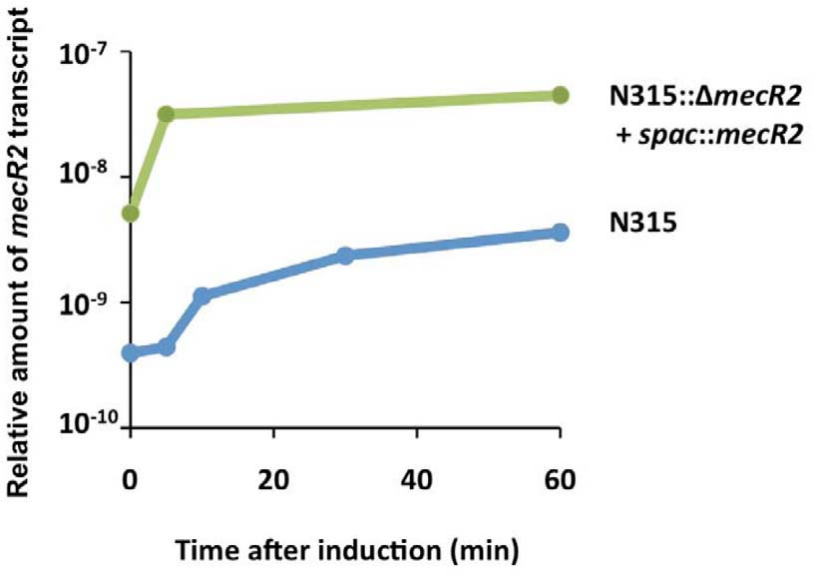
*mecR2* transcription analysis. qRT-PCR analysis of the *mecR2* induction profile in parental strain N315 and its complemented *mecR2* mutant (N315::Δ*mecR2*+*spac*::*mecR2*, IPTG 100 µM). Cultures were induced with a sub-MIC concentration of oxacillin (0.05 mg/L).

We have also analyzed by qRT-PCR the induction profile of *mecR2* in parental strain N315 and in the complemented *mecR2* null-mutant (N315::Δ*mecR2*+spac::*mecR2*) – Figure 6. qRT-PCR data for parental strain N315 showed that *mecR2* transcription was induced in the presence of sub-MIC oxacillin, in agreement with data from RT-PCR that showed that *mecR2* was co-transcribed with *mecR1-mecI* from the inducible *mecR1* promoter (Figure S2). In the complemented mutant, in the presence of the inducer (IPTG), *mecR2* transcription levels were 10 fold higher than those of parental strain and, as such, the low levels of *mecA* transcription observed for this strain (Figure 5) cannot be attributable to an inefficient induction of *mecR2* transcription from the P*spac* promoter.

Of note, in parental strain N315, *mecR2* transcription levels appear to be residual when compared to those of *mecA* (10^9^ fold less). Although this might be explained by an experimental artifact, one can also speculate that this may be due to different promoter strengths and/or to promoter blockage by RNA polymerase, since *mecA* and *mecR1* promoters are divergent and overlap partially. In terms of signal-transduction mechanism, once the expression of *mecA* is induced in response to β-lactams, there is no need for high cellular levels of inducer, repressor or anti-repressor. Actually, the basal transcription of *mecR1-mecI-mecR2* is only necessary to assure that the repressor protein is still present when the antibiotic induction stops, so that the transcription of the resistance gene is shutdown. The apparent very low transcription level of *mecR2* in parental strain N315 may also explain the lack of complementation when *mecR2* was over-expressed *in trans*. In fact, this artificial system, when compared to wild-type strains, presumably generates extremely high cellular amounts of MecR2, which may originate a loss of function by oligomerization or non-specific interactions with other cellular targets. Finally, the apparent residual *mecR2* transcription levels may also explain our failed attempts to analyze by Northern blotting the transcription of *mecR2* in prototype MRSA strains, even with large amounts of total RNA (10–30 µg) and long autoradiograph expositions (72 h). To our knowledge, Northern blotting analysis of *mecR1-mecI*/*blaR1-blaI* transcripts was described in only two studies and, in both cases, clear signals were obtained only when regulatory genes were overexpressed from recombinant plasmids [33], [34].

### *mecR2* is essential for the optimal expression of β-lactam resistance in strains with functional *mecI-mecR1* regulatory locus

Among the five major SCC*mec* types, only SCC*mec* types II and III are characterized by complete *mecR1-mecI* regulatory locus [26] – Figure 1A. SCC*mec* type III strains appear to have a conserved point mutation within *mecI* coding sequence resulting in a truncated non-functional repressor protein [24], [35]. Concerning SCC*mec* type II strains, the accumulation of deleterious mutations has also been described in some strains [19]–[23]. However, data from our MRSA collections [24], as well as from available genomic and SCC*mec* type II sequences, suggest that many strains have wild-type sequences for *mecI* (and *mecR1*). For instance, in a BLAST analysis against the *mecI* sequence from strain N315, c.a. 20 entries were found with 100% sequence identity, mostly from *S. aureus* strains but also from a few coagulase-negative staphylococci (*S. epidermidis*, *S. saprophyticus, S. fleurettii, S. cohinii*, etc.). These observations suggest that the *mecR2* function may be required for the optimal expression of β-lactam resistance in those SCCmec type II strains with wild-type sequences for *mecI and mecR1*.

**Figure 7:**
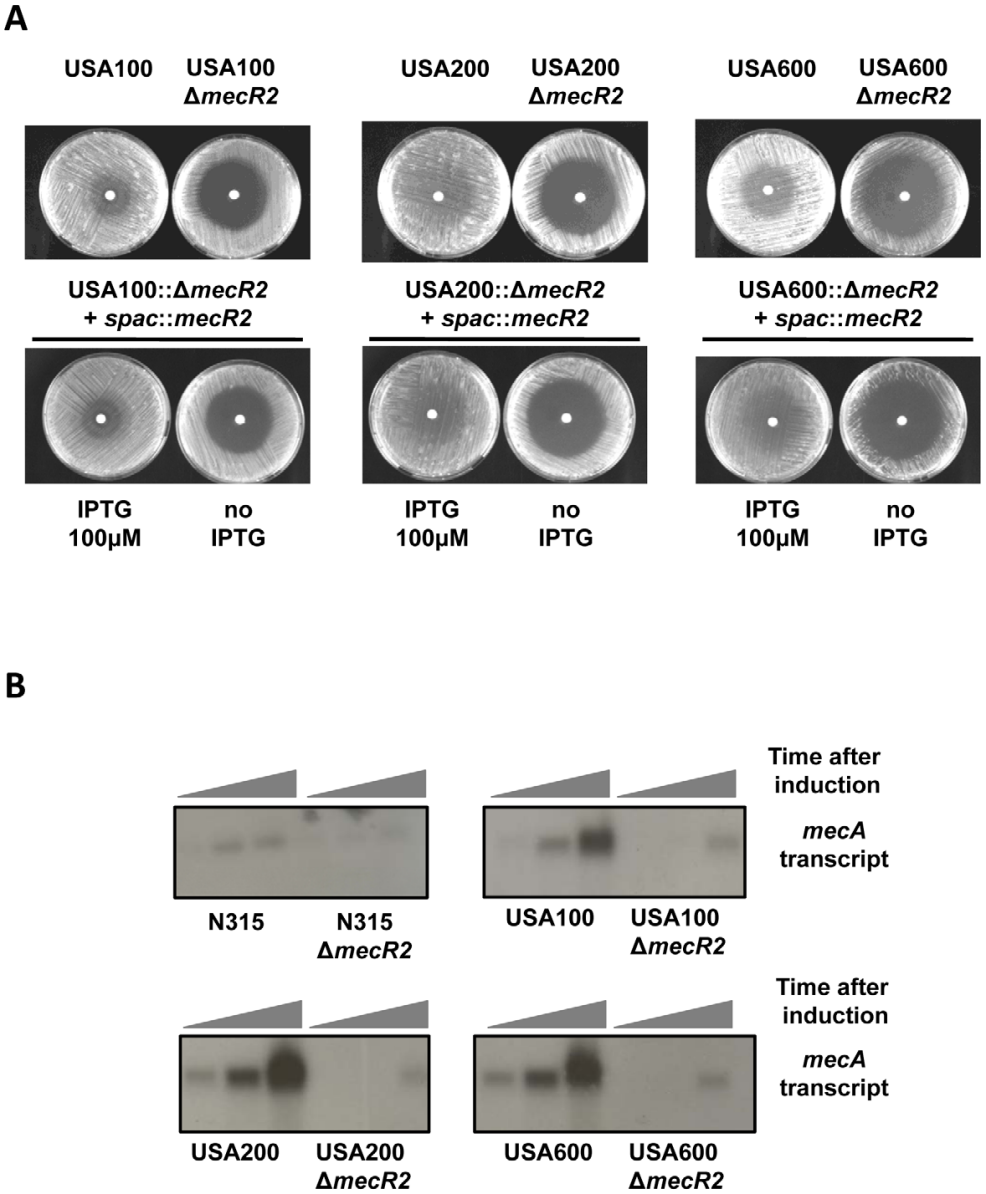
*mecR2* is essential for the optimal expression of β-lactam resistance in strains with functional *mecI-mecR1* regulatory locus. (A) Deletion of *mecR2* from the chromosome of prototype epidemic strains USA100, USA200 and USA600 harboring SCC*mec* type II causes a decrease on the resistance level to oxacillin, which can be reverted upon complementation with *mecR2* expressed from an inducible promoter (*spac*::*mecR2*) in the presence of the inducer (IPTG 100 µM). (B) Northern blot analysis of the *mecA* induction profile in parental strains USA100, USA200 and USA600 and respective *mecR2* null-mutants. Cultures were induced with a sub-MIC concentration of oxacillin (0.05 mg/L) and samples were taken at 0′, 10′ and 60′. For comparative purposes the profile of parental strain N315 and *mecR2* null-mutant were also repeated. Note that film was exposed for 4 h whereas in Figure 5A it was exposed for 48 h.

In order to explore that hypothesis, we sought to test the role of *mecR2* in the phenotypic expression of β-lactam resistance in prototype strains of epidemic MRSA clones characterized by SCC*mec* type II. The MRSA population has a very strong clonal structure and only a few epidemic clones are responsible for the majority of infections worldwide [36], [37]. Three epidemic MRSA clones characterized by SCC*mec* type II have been described [38]: clone ST5-II, “New York/Japan” or USA100; clone ST36-II, EMRSA-16 or USA200; and clone ST45-II or USA600. MRSA clones ST5-II and ST36-II are two of the most important nosocomial clones in the USA and UK, respectively. Prompted by this epidemiological data, we evaluated the role of *mecR2* in three representative strains of those SCC*mec* type II clones selected from a large US collection of MRSA [39]: strains USA100, USA200 and USA600. For this purpose, the chromosomal *mecR2* deletion of strain N315 (N315::Δ*mecR2*) was transduced into those strains originating the recombinant strains: USA100::Δ*mecR2*, USA200::Δ*mecR2* and USA600::Δ*mecR2*. In the three prototype strains, deletion of *mecR2* caused a sharp decrease of the phenotypic expression of β-lactam resistance, which could be complemented by expressing *mecR2 in trans* under the control of an inducible promoter (spac::*mecR2*) (Figure 7A). We have also analyzed the effect of *mecR2* on the induction of *mecA* transcription in strains USA100, USA200 and USA600 by Northern blotting. Compared to N315, the three parental strains expressed *mecA* at higher levels and, in agreement with what was observed for strain N315 (Figure 5), deletion of *mecR2* caused a sharp decrease on the *mecA* induction and transcription levels (Figure 7B).

### *mecR2* function is not dependent on *mecR1* neither on the β-lactamase locus

**Figure 8:**
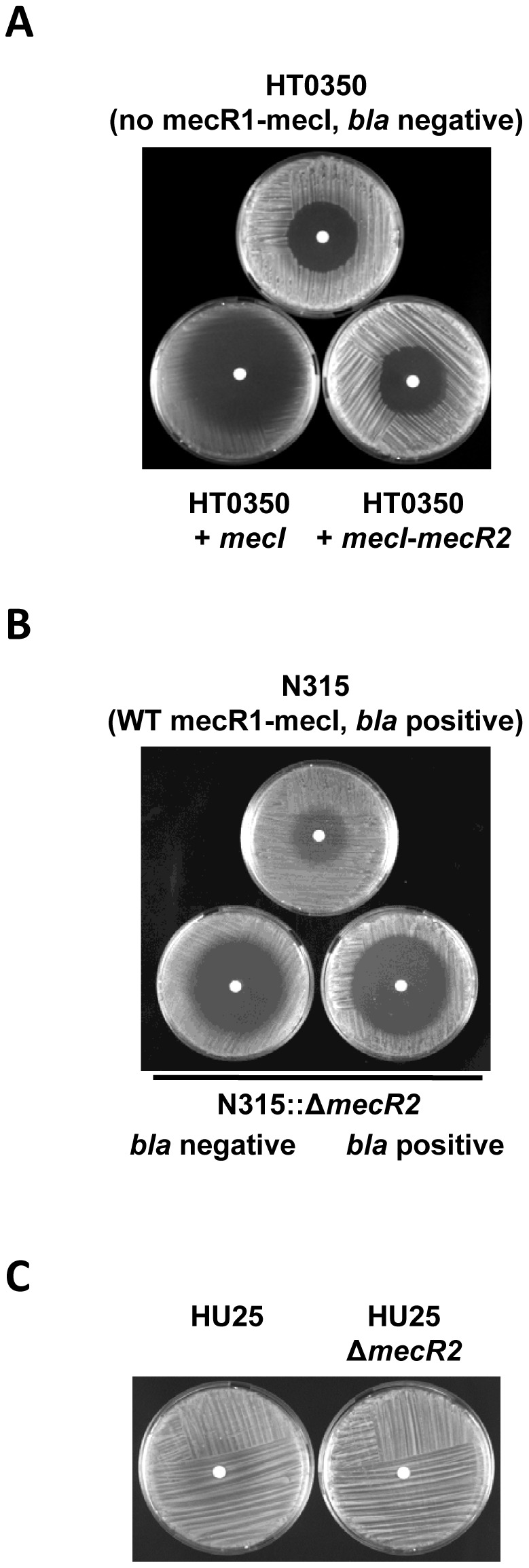
The *mecR2* function is not dependent of *mecR1* neither of the β-lactamase locus. (A) Prototype strain HT0350 is negative for *mecR1-mecI* and for the β-lactamase locus. Co-overexpression of *mecI* and *mecR2* in strain HT0350 (HT0350+*mecI-mecR2*), reverts the effect of *mecI* overexpression (HT0350+*mecI*). (B) The strategy used to delete *mecR2* in prototype strain N315 generated an intermediate mutant that has lost the β-lactamase plasmid. The chromosomal *mecR2* deletion was then transduced back to the parental strain generating a β-lactamase positive *mecR2* null mutant. In both variants, the deletion of *mecR2* caused a sharp decrease of the resistance level to oxacillin. (C) Prototype strain HU25 is positive for *mecR2* and the β-lactamase locus and has a truncated non-functional MecI and, as such, the *mecA* expression is under the exclusive control of *bla* regulatory genes. Deletion of *mecR2* in strain HU25 (HU25::Δ*mecR2*) has no effect on the phenotypic expression of oxacillin resistance.

Since strains N315, USA100, USA200, and USA60 have complete *mecR1* genes and strain COL has a truncated *mecR1* gene but with a complete N-terminal inducer domain, with the previous experiments we could not formally exclude that MecR2 function is dependent of at least the N-terminal inducer domain of MecR1. Therefore, we sought to test the effect of *mecR2* in a prototype SCC*mec* type V MRSA strain, characterized by an extensive deletion of *mecR1* spanning both N- and C-terminal domains [40] – Figure 1A. Among our collections, we selected strain HT0350 [41], since it was the only strain also negative for the β-lactamase locus [24]. Similar to what was observed for strain COL, overexpression of MecI in strain HT0350 (HT0350+*mecI*) caused a sharp decrease of resistance level, which was fully reverted with the co-overexpression of MecI and MecR2 (HT0350+*mecI-mecR2*) – Figure 8A. These data suggests that the effect of *mecR2* on the expression of β-lactam resistance in *S. aureus* is not dependent of *mecR1*, and as such MecR2 may act as an anti-repressor.

Since *mecA* transcription can be co-regulated by the regulators of the β-lactamase (*bla*) locus, *blaR1-blaI*, and parental strains N315, USA100, USA200 and USA600 are *bla* positive, we sought to evaluate the effect of *bla* genes on the observed *mecR2*-induced phenotypes. For this purpose, we took advantage of the fact that the experimental strategy used to construct the *mecR2* knockout in prototype strain N315 generated an intermediate mutant strain which lost the β-lactamase plasmid, probably due to the multiple passages, many of which at 45°C. As in all other chromosomal manipulations, the *mecR2* genetic deletion was transduced back to the parental β-lactamase positive strain N315 to generate the final deletion mutant (N315::Δ*mecR2*) tested in all previous experiments. As illustrated in Figure 8B, in both variants of the *mecR2* chromosomal deletion, there was a sharp decrease of the β-lactam resistance. Together with the experimental data for strains COL and HT0350, both *bla* negative, this assay indicated that the *mecR2* function on the phenotypic expression of β-lactam resistance is not dependent on the presence of the β-lactamase plasmid.

In addition, in order to exclude an interaction of MecR2 with *bla* regulators, we sought to evaluate the phenotype of a *mecR2* deletion mutant in prototype strain HU25, a highly resistant MRSA strain which is positive for the *bla* locus and has a truncated non-functional MecI protein due to a premature stop codon [24]. Previous studies have shown that in the presence of oxacillin, the transcription of *mecA* is readily induced in strain HU25, presumably by the *bla* system [24]. As illustrated in Figure 8C, the absence of *mecR2* in strain HU25 (strain HU25::Δ*mecR2*) had no effect on the phenotypic expression of oxacillin resistance, suggesting that MecR2 is not required for the *mecA* induction mediated by the BlaR1-BlaI system.

### MecR2 interacts directly with MecI

**Figure 9:**
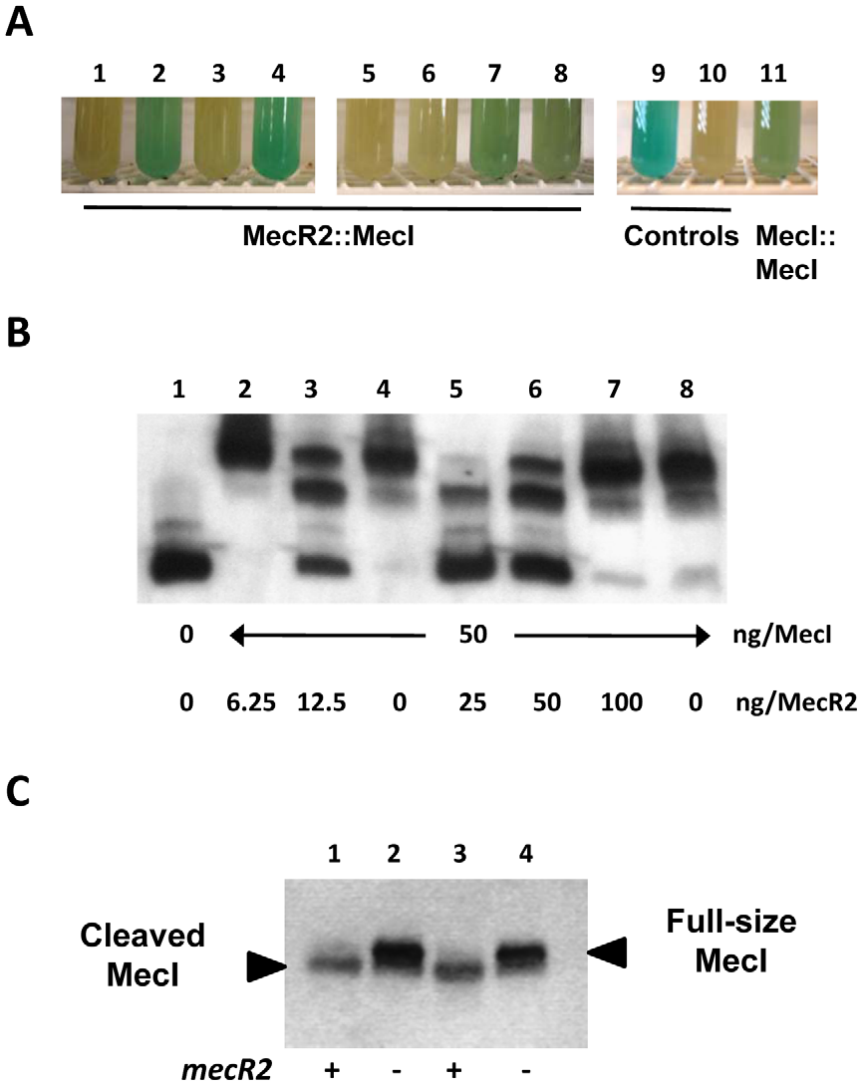
MecR2 interacts directly with MecI, interfering with the binding of MecI to the *mecA* promoter and fostering the proteolysis of MecI. (A) *In vivo* analysis of the MecR2::MecI interaction using the bacterial two-hybrid strategy. This strategy is based on the restoration of the adenylate cyclase (CyaA) activity of *E. coli*, which activates a specific reporter gene, *lacZ*. Interactions between protein fusions were evaluated in liquid cultures through the hydrolysis of the chromogenic X-gal substrate by the activated β-galoctasidase. The MecR2::MecI interaction was evaluated using the eight possible combinations: fusions either with T25 or T18 fragments of CyaA at either the N′ or C′ terminals. Tube 1, T18-MecR2::MecI-T25; tube 2, T18-MecR2::T25-MecI; tube 3, MecR2-T18::MecI-T25; tube 4, MecR2-T18::T25-MecI; tube 5, MecR2-T25::T18-MecI; tube 6, T25-MecR2::T18-MecI; tube 7, MecR2-T25::MecI-T18; tube 8, T25-MecR2::MecI-T18; tube 9, positive control provided by the manufacturer (Zip-T25::Zip-T18); tube 10, negative control (T25::T18); tube 11, “in-house” positive control testing the MecI-MecI interaction T18-MecI::T25-MecI. (B) Electrophoretic mobility shift assay (EMSA) of the binding of purified MecI to a labeled 212 bp DNA fragment encompassing the *mecA* promoter in the presence of purified MecR2. MecI concentration was constant in all binding reactions (0.05 µg). Lane 1, negative control, labeled DNA only; lane 2, 8-fold excess of MecI; lane 3, 4-fold excess of MecI; lane 4, binding control, MecI only; lane 5, 2-fold excess of MecI; lane 6, equimolar amounts of MecI and MecR2; lane 7, 2-fold excess of MecR2; lane 8, control for specific binding, MecI with a 125 molar excess of unlabelled DNA. (C) Western blotting analysis of MecI cleavage in total protein extracts (60–80 mg/lane). Lane 1, prototype strain N315; lane 2, *mecR2* null-mutant (N315::Δ*mecR*2); lane 3, strain HT0350 co-overexpressing MecI and MecR2 (HT0350+*mecImecR2*); lane 4, strain HT0350 overexpressing MecI (HT0350+*mecI*). Cultures of N315 and N315::Δ*mecR2* cultures were induced with a sub-MIC concentration of oxacillin (0.05 mg/L).

The MecR2 is homologous to the transcriptional repressor of the xylose operon, XylR [42], which has a N-terminal DNA-binding domain and a C-terminal dimerization domain. The *mecR2* gene in the prototype strain N315 has no DNA binding domain due to a deletion of four tandem-thymine residues, which, together with the genetic experiments done with this variant (Figures 4, 7 and 8), suggests that only the dimerization domain is involved in the MRSA phenotype. Therefore, we reasoned that the mode of action of MecR2 might involve a direct interaction with the MecI dimer, through its dimerization domain, which eventually would interfere with its binding to the *mecA* promoter. To test this hypothesis, we evaluated the MecR2-MecI interaction using a bacterial two-hybrid *in vivo* strategy [43]. In these experiments, we used the small *mecR2* variant present in prototype strain N315. As in-house controls, the MecI::MecI interaction, previously demonstrated using the yeast two-hybrid strategy [44], as well as the MecR2::MecR2 interaction were also evaluated. Positive results were observed in 4 out of the 8 MecI::MecR2 combinations and in 1 of 4 MecI::MecI combinations (Figure 9A). No MecR2::MecR2 interaction was detected in the four combinations tested (data not shown) and, as such, the assay was not conclusive in this case. Altogether, these observations provide evidence for a MecR2::MecI direct interaction.

### MecR2 interferes with the binding of MecI to the *mecA* promoter

Next, we evaluated the interference of purified MecR2 protein with the binding of MecI to the promoter of *mecA* (P*mecA*) at several molar ratios by the electrophoretic mobility shift assay (EMSA), a strategy previously used to study the binding of purified MecI protein to P*mecA* [14], [24]. In these experiments, we expressed in *E. coli* the full MecR2 protein from prototype strain HU25, since the shorter variant of strain N315 could not be expressed and purified in a soluble form at high concentrations. As illustrated in Figure 9B, MecR2 interferes with the binding of MecI to P*mecA* in a concentration-dependent manner: the heavier band presumably reflecting the binding of MecI dimers to P*mecA* decreases in intensity, whereas the intermediate band reflecting the binding of MecI monomers to P*mecA* and the lighter free DNA band increase in intensity. In line with the genetic experiments, this effect was optimal for a MecR2::MecI molecular ratio below one; in the presence of excess MecR2 the binding of MecI to P*mecA* was restored. This *in vitro* loss of effect at higher concentrations of MecR2 suggests that under these conditions MecR2 may be trapped in a non-active conformation; e.g. MecR2 may oligomerize in a concentration-dependent manner and stop interacting with MecI. It should be noted that in wild-type strains, *mecI* and *mecR2* are co-transcribed from the *mecR1* promoter and, as such, the cellular amounts of both proteins are likely to be similar. Since in these experiments we used the full MecR2 variant containing a putative N-terminal DNA binding, control EMSA experiments with MecR2 alone were performed to verify that purified MecR2 did not bind to P*mecA* alone (Figure S4A). In addition, control experiments with mixtures of MecI and MBP (maltose-binding protein, which has an identical molecular weight to MecR2) were performed to demonstrate that inhibition of MecI binding to P*mecA* is specific for MecR2 (Figure S4B). Finally, in order to exclude the hypothesis that at higher concentrations MecR2 binds (not specifically) to secondary sites in P*mecA* DNA in a MecI-dependent manner, EMSA assays with MecI-MecR2 mixtures were also performed with a much smaller DNA fragment (39 bp instead of 212 bp) containing the MecI protected sequences and the same results were obtained (data not shown). Altogether, these assays demonstrate that MecR2 acts as an anti-repressor disturbing the binding of MecI to the *mecA* promoter.

### MecR2 promotes the proteolytic cleavage of MecI

Based on structural data, it has been suggested that the proteolysis of MecI observed during *mecA* induction is not mediated by the activated MecR1 inducer domain and instead, is a secondary event not required for induction [45]–[47]. This is in agreement with what has been found for the β-lactamase system of *Bacillus licheniformis* [48]. We speculated that MecR2, by interacting with MecI and disturbing its binding to the *mecA* promoter, could foster a local melting of MecI-dimers, making the scissile bonds more accessible to proteolytic inactivation. To test this hypothesis, we compared by Western blotting the MecI proteolysis in total protein extracts from prototype strain N315 *versus* its *mecR2* null-mutant under induction conditions, and from strain HT0350 overexpressing MecI-MecR2 *versus* HT0350 overexpressing MecI only. As illustrated in Figure 9C, in the absence of MecR2, intact MecI accumulates in both pairs of strains analyzed. Because parental strain HT0350 is negative for all *mecA* regulators and its derivatives used in these experiments overexpress constitutively MecI-MecR2 or MecI, the observed MecR2-induced proteolysis of MecI does not involve MecR1 (neither BlaR1) and, most likely, is mediated by unspecific cytoplasmatic proteases.

### MecR2, the missing link in the signal-transduction mechanism of *mecA* expression

The findings described in this report clarify some critical aspects of the unique signal transduction mechanism underlying the induction of *mecA* gene.

First, we demonstrated that the cognate *mecA* regulatory locus contains, besides MecR1-MecI, the anti-repressor MecR2. MecR2 compensates for the inefficient MecR1-mediated induction of *mecA*, being essential for the optimal expression of β-lactam resistance (Figures 3, 4 and 7A), and enabling the full induction of *mecA* transcription (Figures 5 and 7B). These findings explain the puzzling observation of the poor *mecA* induction by MecR1, reported in studies analysing the effects of *mecR1-mecI* only (without *mecR2*) on *mecA* expression in recombinant strains [12], [13]; an experimental artefact also observed in this study with recombinant strain COL::RI (artificially made positive for *mecR1-mecI* only) and in the *mecR2* null mutant strains (Figures 4 and 7). Because wild-type MRSA strains positive for *mecR1-mecI* are also positive for *mecR2*, these strains are in fact able to express optimal levels of β-lactam resistance and, as such, *mecA* is efficiently induced upon exposure to β-lactams by its cognate three-component regulatory system.

Second, the findings herein described also clarify the relevance and specificity of MecI proteolysis observed upon induction with β-lactams [16], [45]–[48]. Our data demonstrates that MecI proteolysis is required for optimal expression of resistance and that MecR2 alone (i.e. without MecR1, Figure 8A) interferes specifically with the MecI function and promotes its inactivation by proteolytic cleavage, presumably mediated by (non-specific) native cytoplasmatic proteases (Figure 9). Our findings contrast with published observations for the *blaR1-blaI* system of *Bacillus licheniformis*, demonstrating that the proteolysis of the repressor is a secondary event not required for induction of resistance [48], and also for the *blaR1-blaI* system of *S. aureus* demonstrating that BlaR1 directly promotes the BlaI cleavage [15]. Altogether, these observations suggest the existence of subtle differences between the *mecR1-mecI-mecR2* and the *blaR1-blaI* regulatory systems and that, in *S. aureus*, BlaR1 may accumulate the MecR1 and MecR2 functions.

**Figure 10:**
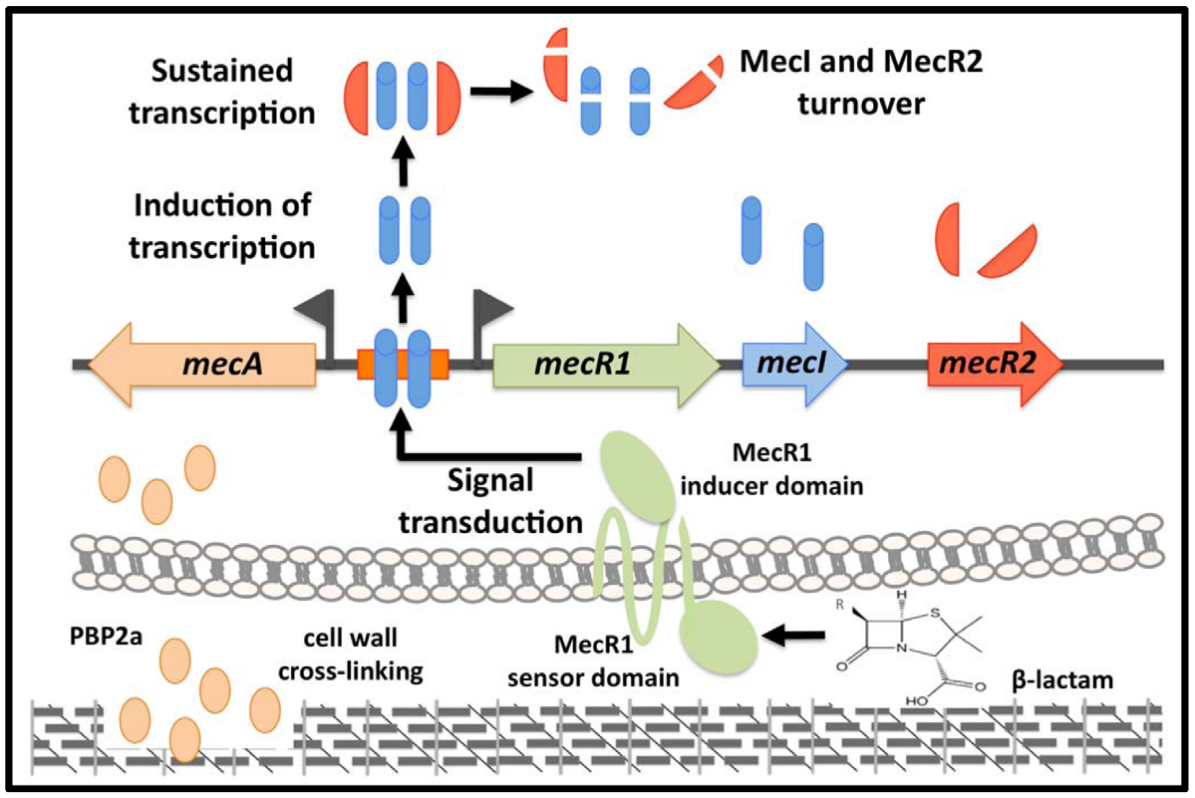
Model for the *mecA* induction by MecR1-MecI-MecR2. In the presence of a β-lactam antibiotic, MecR1 is activated and rapidly induces the expression of *mecA* and *mecR1-mecI-mecR2*. The anti-repressor activity of MecR2 is essential to sustain the *mecA* induction since it promotes the inactivation of MecI by proteolytic cleavage. In the absence of β-lactams, MecR1 is not activated and a steady state is established with stable MecI-dimers bound to the *mecA* promoter and residual copies of MecR1 at the cell membrane.

Our findings lead us to propose a revised model for the induction of *mecA* expression in wild-type MRSA strains (Figure 10): (i) in the presence of β-lactams, MecR1 is activated and induces the transcription of *mecA* and *mecR1-mecI-mecR2*; (ii) the anti-repressor MecR2, destabilizes MecI-dimers, disturbing their binding to the *mecA* promoter and fostering their proteolytic inactivation, resulting in a sustained induction of *mecA* transcription; (iii) when depletion of β-lactam occurs, MecR1 is no longer activated and a steady state is established consisting of stable MecI-dimers bound to the *mecA* promoter (and protected from proteolysis) and residual copies of MecR1 at the cell membrane; the remaining free MecR2 molecules are most likely degraded by the cellular protein turnover pathway. This model implies that in the absence of MecR2, functional MecI-dimers are more resilient to proteolytic inactivation and outcompete the MecR1 signalling, a hypothesis supported by the Western blotting experiments (Figure 9C).

### Concluding remarks

This study demonstrates that the central element of methicillin-resistance in *S. aureus*, the *mecA* gene, can be regulated by a three-component system consisting of a transcriptional repressor, a sensor-inducer and an anti-repressor, a very unusual arrangement for the transcriptional control of genes in bacteria. In addition, the induction of the resistance gene expression involves a unique series of proteolytic steps, being the proteolytic cleavage of the repressor modulated by the anti-repressor.

This study also sheds light on the evolution of antibiotic-resistance genes. The *mecA* gene itself is probably ancient and predates the use of antibiotics in clinical practice [49], [50]. Before its recent acquisition by MRSA, *mecA* was assembled into a gene complex containing its transcriptional regulators and incorporated into a mobile genetic element. Tsubakishita *et al.* have proposed that the *mecA* gene complex found in MRSA has been assembled in the animal-related *Staphylococcus fleurettii* species [51]. Remarkably, in this species the *mecA-mecR1-mecI* locus was found immediately upstream to the complete and functional xylose operon, containing the XylR repressor homologous to MecR2. This suggests that a specific selection acted on XylR, a transcriptional repressor of sugar metabolism, to originate the MecR2 function, an anti-repressor of an antibiotic-resistance gene, and that the three-component *mecA* regulatory locus was assembled in *S. fleuretti* before being transferred to *S. aureus*.

In short, this study points to a revision of the model for the transcriptional control of *mecA* by its cognate regulatory locus, which may pave the way for the design of alternative therapeutic strategies targeting the induction mechanism of the resistance gene [52], [53]. If successful, these strategies may extend the clinical utility of β-lactams for the treatment of MRSA infections. Recycling β-lactams is particularly relevant given that MRSA pose a substantial burden for the public health, are often multi-drug resistant and, in the past 40 years, very few new classes of antibiotics have reached the clinic.

## Materials and Methods

### Bacterial strains and growth conditions

The bacterial strains and plasmids used in this study are listed in Tables S1 and S2, respectively. *S. aureus* strains were routinely grown at 37°C with aeration in tryptic soy broth (TSB, Difco) or on tryptic soy agar plates (TSA, Difco). *E. coli* strains were grown with aeration at 37°C in Luria-Bertani broth (LB, Difco) or in Luria-Bertani agar (LA, Difco). Recombinant *E. coli* strains were selected and maintained with ampicillin at 100 µg/mL. Recombinant *S. aureus* strains were selected and maintained either with tetracycline at 5 or 40 µg/mL, chloramphenicol at 20 µg/mL, or erythromycin at 10 µg/mL, as appropriate. Phenotypic analysis of β-lactam resistance in *S. aureus* parental and recombinant strains was performed by diffusion-disks containing 1 mg of oxacillin, Oxacillin E-test (AB Biodisk), or by population analysis profiles (PAPS) at 30°C for 24–48 h, as previously described [24], [54], [55]. Oxacillin is a methicillin analogue and has replaced methicillin in clinical use.

### DNA manipulations

DNA manipulations were performed by standard methods [56], [57]. Total DNA from *S. aureus* was isolated from bacterial cultures with the Wizard Genomic DNA purification Kit (Promega) according to the manufacturer's recommendations and using lysostaphin (0.5 mg/mL) and RNAse (0.3 mg/mL) in the lysis step. Plasmid DNA was isolated from bacterial cultures with the High Pure Plasmid Isolation Kit (Roche). For plasmid DNA isolation from *S. aureus* strains the culture pellets were ressuspended in “Suspension Buffer” supplemented with 0.1 mg/mL of lysostaphin and incubated at 37°C for 30–60 minutes. Restriction enzymes were used as recommended by the manufacturer (New England Biolabs). Dephosphorilation of vector arms and insert ligation was performed with Rapid DNA Dephos & Ligation kit (Roche), according to the manufacturer's recommendations. Routine PCR was performed with GoTaq Flexi DNA polymerase (Promega). PCR amplification of cloning inserts was performed by high-fidelity PCR (*Pfu* Turbo DNA polymerase, Strategene). DNA purification from PCR and digestion reactions was performed with High Pure PCR Product Purification Kit (Roche). For ligation protocols, the inserts and linearized plasmids were resolved in a low melting agarose gel (1%) (Invitrogen) and DNA bands were purified with Gene Clean Turbo kit (MP Biomedicals), following the manufacturer's recommendations. DNA sequencing was performed by Macrogen (www.macrogen.com) or STAB Vida (www.stabvida.com). All primers used in this study are listed in Table S3.

### Construction of recombinant *S. aureus* strains

All recombinant plasmids used in this work were firstly constructed and stabilized in *E. coli* DH5α, electroporated into *S. aureus* restriction-deficient strain RN4220 and finally transduced by the 80α phage to the desired parental strain, as previously described [58], [59]. The Integrity of plasmid inserts was confirmed by restriction analysis, PCR and DNA sequencing. The integrity of chromosomal insertion-deletions was confirmed by PCR, DNA sequencing and Southern blotting of pulsed-field gel electrophoresis of chromosomal DNA. Chromosomal insertion-deletions were backcrossed by phage transduction to the original parental strains.

To co-overexpress *mecI* and *mecR2* in strain COL, a fragment containing the *mecI-mecR2* region from strain N315 was amplified using primers MI-P1/MR2-P1, double-digested with EcoR1/BamH1 and cloned into pGC2, originating the recombinant plasmid pGC::*mecI-mecR2*. To reconstruct the *mecA* regulatory locus in the chromosome of strain COL, we first construct pSPT::IS-*erm*, a pSPT181 derivative containing the terminal fragment from IS*1272* located in the upstream vicinity of *mecA* in strain COL and the erythromycin (*erm*) resistance cassette gene from Tn*551*. The 0.6 kb terminal fragment of IS*1272* was amplified from strain COL using primers IS1272-P1/IS1272-P2, double-digested with PstI/SalI and cloned into pSPT181, originating pSPT::IS. The *erm* cassette was recovered from the pSP64E plasmid by BamHI/SalI double-digestion and was cloned into pSPT::IS, originating pSPT::IS::*erm*. To reconstruct the *mecR1-mecI* locus in strain COL (strain COL::RI), we amplified by high-fidelity PCR a 1.9 kb DNA fragment from strain N315 chromossomal DNA, containing the wild-type coding sequences of *mecR1* and *mecI* genes, using primers MI-P2/MR1-P1. The fragment was double-digested with BamH1/AvaI and directionally cloned into pSPT::IS-*erm*, originating pSPT::IS-*erm*-*mecI-mecR1*. To reconstruct the full *mecA* regulatory locus in strain COL (strain COL::RI-R2), we amplified by high-fidelity PCR a 3.5 kb DNA fragment from strain N315 chromossomal DNA, containing the *mecR1-mecI-mecR2* locus, using primers MR2-P1/MR1-P1. The fragment was double-digested with BamH1/AvaI and directionally cloned into pSPT::IS-*erm*, originating pSPT::IS-*erm-mecR2-mecI-mecR1*. As control, we constructed a strain with a integrated *erm* gene in the *mecA* upstream vicinity (strain COL::*erm*): a 0.5 kb DNA fragment containing the terminal fragment of the N-terminal cytoplasmatic domain of *mecR1*, was amplified using primers MR1-P1/MR1-P2, double-digested with BamH1/AvaI and cloned in pSPT::IS-*erm*, originating pSPT::IS-*erm*-Δ*mecR1*. The integration into COL chromosome of the three recombinant plasmids (pSPT::IS-*erm*-*mecI-mecR1*, pSPT::IS-*erm*-*mecR2-mecI-mecR1* and pST::IS*-erm*-Δ*mecR1*) was performed by an insertion-deletion strategy by homologous recombination (Figure S3). First, insertion into the chromosome was promoted by growing transductants in TSB at a non-permissive temperature (45°C) without antibiotic selection for 2–3 days, with daily re-inoculum in fresh medium. Serial dilutions were plated onto TSA plates supplemented with erythromycin (Ery) and tetracycline (Tc). Single-colonies Erm^r^-Tc^r^ were screened for chromosomal insertion of the plasmids by PCR and the absence of plasmid DNA was confirmed. Resolution of integrates by homologous-recombination was promoted by growing selected single colonies in TSB supplemented with tetracycline at 40 µg/mL at the permissive temperature of 30°C for 4–5 days, with daily re-inoculum in fresh medium. Finally segregation of the excised plasmids was promoted by growing cultures at 45°C without antibiotic selection for 2–3 days, with daily re-inoculum in fresh medium. Culture aliquots were plated onto TSA plates supplemented with erythromycin and single colonies Erm^r^-Tc^s^ were selected by replica plating onto TSA plates supplemented with erythromycin or tetracycline.

To construct the *mecR2* gene null mutant in strain N315, two DNA fragments of 1000 bp corresponding to the 5′ and 3′ vicinities of the *mecR2* gene were amplified by PCR from strain N315 DNA using primers MR2-P2/MR2-P3 and MR2-P4/MR2-P5, respectively. The *cat* gene coding for chloramphenicol resistance was also amplified by PCR from pGC2 plasmid with primers CAT-P1/CAT-P2. The three fragments were double-digested with SalI/PstI, BamH1/XhoI and XhoI/SalI, respectively, and then sequentially cloned into pSPT181, originating the pSPT::*cat*-Δ*mecR2* recombinant plasmid. Following the same insertion-deletion strategy described above, but selecting for chloramphenicol resistance instead of erythromycin resistance, we obtained the recombinant strain N315::Δ*mecR2* in which the chromosomal copy of *mecR2* was replaced by the *cat* gene (Figure S3). To complement the N315Δ*mecR2* null-mutant three recombinant plasmids were constructed: (i) pSPT::*mecR2*, pSPT181 vector containing at the XmaI site the *mecR2* gene from strain N315, obtained by PCR with primers MR2-P6/MR2-P7 (the proper insert orientation was selected by restriction analysis using the HindIII site within *mecR2* gene); (ii) pSPT::*mecImecR2*, pSPT181 containing the *mecI-mecR2* genes of strain N315, constructed by sequential cloning, first, at the BamH1/PstI, the *mecI* gene site obtained with primers MI-P3/MI-P4 and then, at the XmaI site, the *mecR2* gene obtained with primers MR2-P6/MR2-P7; (iii) pSPT::*spac*-*mecR2*, pSPT181 with the *mecR2* gene under the control of *Pspac* promoter, constructed by sequential cloning the 1.6 kb EcoR1-BamH1 fragment from plasmid pDH88 containing the *spac* locus (P*spac*-polylinker-*lacI* repressor) and then, at the XmaI site of the *spac* polylinker, the *mecR2* gene from strain N315 obtained with primers MR2-P6/MR2-P7. As control, N315Δ*mecR2* was transformed with a pST181 derivative containing the *spac* locus only (pSPT::*spac*).

To generate the *mecR2* gene null mutant in prototype strains USA100, USA200, USA600, and HU25 the chromosomal deletion of strain N315::Δ*mecR2* was transduced by bacteriophage infection with selection for chloramphenicol resistance, originating recombinant strains USA100::Δ*mecR2*, USA200::Δ*mecR2*, USA600::Δ*mecR2* and HU25::Δ*mecR2*. Mutant strains USA100::Δ*mecR2*, USA200::Δ*mecR2* and USA600::Δ*mecR2* were then complemented with recombinant plasmid pSPT::*spac*-*mecR2*.

### Transcription analysis

Total RNA extraction and purification was performed as previously described [60]. Briefly overnight cultures were grown in TSB, supplemented with antibiotics when appropriate, and then diluted 1∶50 in fresh TSB and grown to the mid-log phase (OD_620_∼0.7). Cultures were stabilized with two volumes of RNAprotect Bacteria Reagent (Qiagen), according to the manufacturer's recommendations. The cells were centrifuged and pellets were ressuspended in 1 mL of Trizol reagent (Invitrogen). The ressuspended cells were transferred to a new tube with silica beads (Lysing Matrix B tubes, Bio101) and cell lysis was performed in the FastPrep FP120 apparatus (Bio 101). RNA was extracted with chloroform, precipitated with isopropanol, washed twice with ethanol at 80% and ressuspended in diethyl pyrocarbonate (DEPC)-treated water. For the analysis of the *mecA* and *mecR2* induction profiles, after cultures were grown to OD_620_∼0.7, oxacillin at 0.05 µg/mL was added and cultures were incubated for an additional 60 minutes. Samples were taken either at 0, 5, 15, 30, and 60 or at 0, 10 and 60 minutes, stabilized, pelleted and kept on ice until being simultaneously processed. For RT-PCR and qReal-time RT-PCR experiments (see below), total RNA preps were treated twice with DNAse (RNase-Free DNase Set I, Qiagen) and purified with RNeasy Mini Kit (Qiagen), according to the manufacture's recommendations. Control PCR reactions were performed to test the absence of DNA contamination in total RNA preps.

Transcription analysis of *mecR1-mecI-mecR2* was performed by RT-PCR for mid-log phase induced cultures (oxacillin at 0.05 µg/mL) of strains N315 and HU25 with primer pairs MR2-RT1/MR2-RT2 (*mecR2* transcript), MI-P5/MR2-P8 (*mecI-mecR2* co-transcript), MR1-P3/MI-P6 (*mecR1-mecI* co-transcript), and MA-P1/MA-P2 (*mecA* transcript, inducible positive control). RT-PCR reactions were set-up using the One-Step RT-PCR kit (Qiagen), according to the manufacture's recommendations. To control the absence of DNA contamination, all samples were tested in a parallel reaction without the reverse-transcription step. To control the size of the amplified transcripts, PCR reactions with chromosomal DNA were also performed in parallel. The *mecA* transcript was detected in both induced and non-induced samples suggesting that the RT-PCR assay was too sensitive to discriminate between basal and induced transcription levels.

The induction profiles of *mecA* and *mecR2* were determined by quantitative Real-time RT-PCR (qRT-PCR) and/or Northern blotting. For the qRT-PCR data analysis, relative gene expression was expressed as a ratio to the transcript of *pta*, a housekeeping gene with constitutive expression [61]. Standard curves were generated using serial dilutions (0.4–40 ng/reaction) of genomic DNA and primers MR2-RT3/MR2-RT4, MA-RT1/MA-RT2 and pta-RT1/pta-RT2 for amplification internal fragments of *mecR2*, *mecA*, and *pta*, respectively. qRT-PCR reactions were performed with QuantiTect SYBR Green RT-PCR Kit (Qiagen); each 25 µl reaction containing 12.5 µl SybrGreen mix, 0.25 µl RT enzyme mix, 12.5 pmol of each primer and 40 ng of purified RNA. Amplification consisted of an initial RT step at 50°C for 30 min, followed by a denaturation step at 95°C for 15 min, then by 45 cycles of 30 s at 94°C, 30 s at 53°C and 30 s at 72°C. For each RNA sample three independent qReal-Time RT-PCR experiments were carried out. Fluorescence was measured at the end of the annealing-extension phase of each cycle. A threshold value for the fluorescence of all samples was set manually. The reaction cycle at which the PCR product exceeds this fluorescence threshold was identified as the threshold cycle (C_T_). The C_T_ was then converted to relative quantity of mRNA by using a standard curve. To verify the specificity of the PCR amplification products, melting curve analysis was performed between 60–95°C.

For Northern blot analysis, total RNA (5 µg) was resolved through a 1.2% agarose-0.66 M formaldehyde gel in MOPS (morpholine propanesulfonic acid) running buffer (Sigma). Blotting of RNA onto Hybond N+ membranes (Amersham) was performed with Turboblotter alkaline transfer systems (Schleicher & Schuell). For detection of *mecA* specific transcripts, a DNA probe was constructed by PCR amplification with primers MA-P1 and MA-P2. After purification the probe was labeled with a Ready To Go labeling kit (Amersham) by using [a-^32^P]dCTP (Amersham) and was hybridized under high-stringency conditions. The blots were subsequently washed and autoradiographed.

### Bacterial two-hybrid assays

This strategy is based on the restoration of the adenylate cyclase (CyaA) activity by heterodimerization of protein fusions containing the T25 and T18 fragments, which form the catalytic domain of CyaA. CyaA is involved on cAMP synthesis, which binds to CAP forming the cAMP/CAP complex that activates a specific reporter gene, *lacZ* [43]. All strains and plasmids used in the bacterial two-hybrid studies are described in Table S4. Both genes, *mecI* and *mecR2*, were amplified from the chromosomal DNA of strain N315 by high-fidelity PCR, using primers MI-BTH1/MI-BTH2 for *mecI* and MR2-BTH1/MR2-BTH2 for *mecR2*. PCR products were double-digested with KpnI/XbaI and fused to T25 or T18 fragments either at the N′ or C′ terminals, using plasmids pUT18, pUT18c, pKNT25 and pKT25, originating the following fusion proteins: T18-MecI, MecI-T18, T25-MecI, MecI-T25, T18-MecR2, MecR2-T18, T25-MecR2 and MecR2-T25. The eight MecI::MecR2 recombinant plasmid combinations were co-transformed into the reporter strain *Escherichia coli* BTH101 and grown on Luria-Bertani (LB) and LA agar supplemented with 8 µg/mL 5-bromo-4-choro-3-indolyl-β-D-galactopyranoside (X-gal), 50 µg/mL kanamycin, 100 µg/mL ampicillin, 100 µg/mL streptomycin, 500 µM (IPTG) and 2% glucose. As a positive control, plasmids p25Zip and p18Zip, containing two leucine zipper domains, were also co-transformed into *E.coli* BTH101 strain. Additionally, as in-house controls, the previously reported MecI::MecI interaction based on the yeast two-hybrid strategy [44] was evaluated, as well the MecR2-MecR2 interaction, by co-transforming the four combinations of *mecI*-containing plasmids and the four combinations of *mecR2*-containing plasmids, respectively.

### Electrophoretic mobility shift assays (EMSA)

To overexpress and purify MecR2 protein, a *mecR2* gene insert was obtained from the chromosome of strain HU25 by high-fidelity PCR amplification with primers MR2-cri1/MR2-cri2 and double-digestion with NcoI/XhoI. The *mecR2* insert was then cloned in frame into the expression vector pCri8a, generating the recombinant plasmid pCri8a::*mecR2*, expressing *mecR2* with a N′ terminal His_6_ tag. pCri8a::*mecR2* was stabilized in *E. coli* DH5α and then transformed to *E. coli* Bl21 (DE3). MecR2 protein overexpression was carried out in LB medium supplemented with 50 mg/mL kanamycin, at 18°C, and induced at an OD_600_∼0.5 with 1 µM Isopropyl β-D-1-thiogalactopyranoside (IPTG) for 5 h. MecI protein was overexpressed from recombinant strain DH5α+pP_RO_EX::*mecI* [62], using LB medium supplemented with 100 µg/mL ampicillin, at 37°C and induced at an OD_600_∼0.5 with 1 µM IPTG for 3 h. Protein extracts were purified as previously described [62]. The purity of the proteins was assessed by 10% tricine SDS-PAGE analysis and mass-spectroscopy. The concentrations of purified MecR2 and MecI were estimated using the Protein Assay Kit II (BioRad), as recommended by the manufacturer. For the electrophoretic mobility shift assay we used the chemiluminescent-based DIG Gel Shift Kit (Roche), following the manufacturer's recommendations. As DNA target we used a 212 bp fragment encompassing the *mecA* promoter and operator sequences from prototype strain N315 obtained by PCR amplification with primers MA-PF1/MA-PR1. The binding of each purified protein to the *mecA* promoter (P*mecA*) was first evaluated and then MecI-MecR2 mixtures were tested. As control the binding to P*mecA* of MecI-MBP (Maltose-binding protein, MBP2*, New England Biolabs) mixtures were also evaluated. EMSA assays with MecI-MecR2 mixtures were also performed with a smaller 39 bp DNA fragment containing the MecI protected sequences, obtained by annealing primers MI-Box1/MI-Box2.

### Western blotting

To prepare protein extracts of *S. aureus*, parental and recombinant strains were grown in TSB supplemented with oxacillin at sub-MIC concentration (0.05 µg/mL) until mid-log phase (OD_620_∼0.7). Cell pellets were frozen in liquid nitrogen, thawed and resuspended in Buffer A (50 mM Tris-HCl; 10 mM MgCl2; 0.5 mM PMSF) containing 10 µg/mL DNase I. Cells were broken mechanically in a French press followed by centrifugation (22,000× g, 20 min, 4°C) to remove unbroken cells and cell debris. The supernatants containing the cytoplasmic proteins were recovered and filtered through 0.45-µm-pore-size membrane filters. Protein extracts (60–80 µg) were resolved in a 18% Tris-Glycine SDS-PAGE.

After electrophoresis, the proteins were transferred to a 0,45 µm nitrocellulose membrane (Trans-Blot, Bio-Rad). The membranes were blocked at room temperature for 1 hour, in 20 mL of Blocking solution - Tween- Phosphate Buffered Saline (137 mM NaCl; 2.7 mM KCl; 4.3 mM Na2HPO4; 1.47 mM KH2PO4; 0.05% Tween-20) with 6% low-fat milk. MecI protein was detected by imunoblot analysis using a custom polyclonal antibody (Eurogentec) raised against purified MecI (1/1.000 dilution) and a 1/50.000 dilution of secondary antibody (Goat Anti-Rabbit IgG (H+L) Peroxidase Conjugated Antibody, Pierce) in 10% Blocking solution. The immune complexes were detected using an enhanced chemiluminescence system (SuperSignal West Pico Chemiluminescent; Pierce), according to the manufacturer's instruction. Membranes were exposed to Amersham Hyperfilm ECL film (GE Healthcare).

## Supporting Information

Figure S1 **Multiple sequence alignment between the repressor of the xylose operon (XylR) and the anti-repressor MecR2 found in staphylococci.** Species names (strain code in parenthesis): Sxy – *S. xylosus*, Svi – *S. viridians*, Ssc – *S. sciuri*, Sau – *S. aureus*, Spi – *S. pseudintermedius*, Sfl – *S. fleuretti*, Sep – *S. epidermidis*. Green – identical residues; red – similar residues; white – divergent residues. The figure was prepared using “The Sequence Manipulation Suite” freely available at http://www.bioinformatics.org.
(TIF)

Figure S2 **Transcriptional analysis of** ***mecR2*** **.** (A) Genetic organization of the *mecA* regulatory locus and location of primers used in the RT-PCR assays. (B) Gel electrophoresis of the RT-PCR products obtained with total RNA from strains N315 and HU25, respectively. MM, molecular weight marker (1 Kb DNA ladder); lanes 1–2, *mecI-mecR2* co-transcript; lanes 3–4, *mecR1-mecI* co-transcript; lanes 5–6, *mecR2* transcript; lanes 7–9, positive controls, PCR reactions using the same primer pairs and chromosomal DNA from strain N315 as template; lanes 10–11, negative control, RT-PCR reactions without the reverse-transcription step for total RNA preparations of strains N315 and HU25, respectively, using the primer pair MR2-RT1/MR2-RT2 (i.e. the one originating the smallest amplicon).
(TIF)

Figure S3 **Insertion-deletion strategies used for the reconstruction of the** ***mecA*** **regulatory locus in the chromosome of strain COL and deletion of** ***mecR2*** **from the chromosome of strain N315.** Integration through homologous recombination of recombinant thermosensible plasmids was promoted at a non-permissive temperature (45°C) and with selection for tetracycline resistance (Tc^r^). Resolution of co-integrates was promoted at a permissive temperature (30°C) with selection for tetracycline resistance and segregation of the excised plasmids was promoted at 45°C without antibiotic selection. Colonies susceptible to tetracycline (Tc^s^) and resistant to erythromycin (Ery^r^) or chloramphenicol (Cm^r^) were selected for further analysis.
(TIF)

Figure S4 **Control experiments for the electrophoretic mobility shift assays.** (A) Binding of purified MecR2 to *mecA* promoter. Lane 1, negative control, labeled DNA only; lane 2, 0.001 µg of MecR2; lane 3, 0.01 µg of MecR2; lane 4, 0.05 µg of MecR2; lane 5, 0.1 µg of MecR2; lane 6, 0.25 µg of MecR2; lane 7, 0.5 µg of MecR2; lane 8, 1 ug of MecR2. (B) Binding of purified MecI to the labeled *mecA* promoter DNA sequence in the presence of MBP (Maltose-binding protein) at several molar ratios. MecI concentration was constant in all binding reactions (0.05 µg). Lane 1, negative control, labeled DNA only; lane 2, 8-fold excess of MecI; lane 3, 4-fold excess of MecI; lane 4, binding control, MecI only; lane 5, 2-fold excess of MecI; lane 6, equimolar amounts of MecI and MBP; lane 7, 2-fold excess of MBP; lane 8, control for specific binding, MecI with a 125 molar excess of unlabelled DNA.
(TIF)

Table S1 **Strains used in this study.**
(DOC)

Table S2 **Plasmids used in this study.**
(DOC)

Table S3 **Primers used in this study.**
(DOC)

Table S4 **Strains and plasmids used in the bacterial two-hybrid assays.**
(DOC)
