# Supplementary figures and images for: The Anti-Repressor MecR2 Promotes the Proteolysis of the mecA Repressor and Enables Optimal Expression of β-lactam Resistance in MRSA
Source: PLoS Pathog. 2012 Jul 26;8(7):e1002816. doi: 10.1371/journal.ppat.1002816 (PMC3406092; doi:10.1371/journal.ppat.1002816)

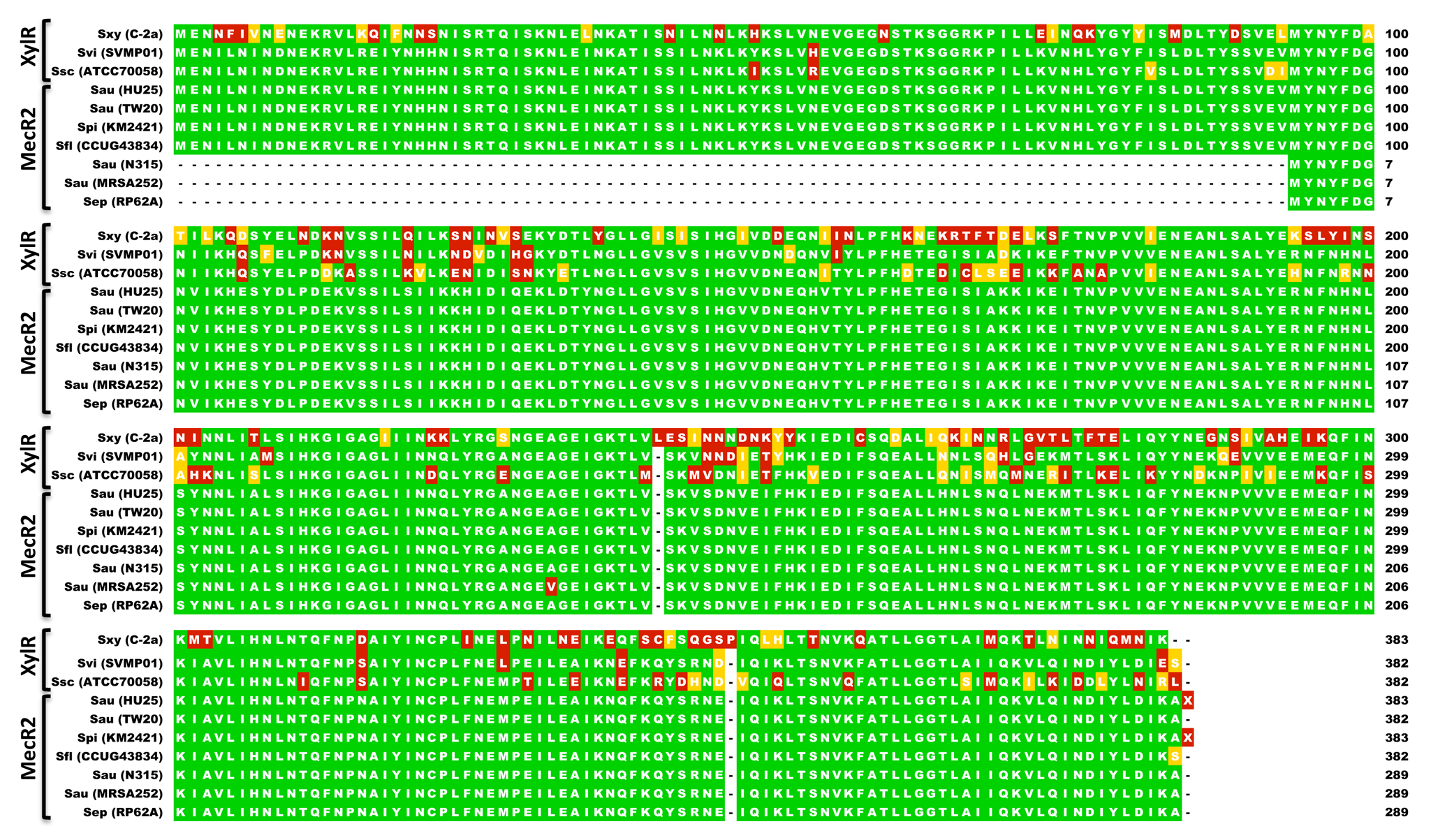

Supplement: Figure S1 — Multiple sequence alignment between the repressor of the xylose operon (XylR) and the anti-repressor MecR2 found in staphylococci. Species names (strain code in parenthesis): Sxy – S. xylosus, Svi – S. viridians, Ssc – S. sciuri, Sau – S. aureus, Spi – S. pseudintermedius, Sfl – S. fleuretti, Sep – S. epidermidis. Green – identical residues; red – similar residues; white – divergent residues. The figure was prepared using “The Sequence Manipulation Suite” freely available at http://www.bioinformatics.org. (TIF) [file ppat.1002816.s001.tif]

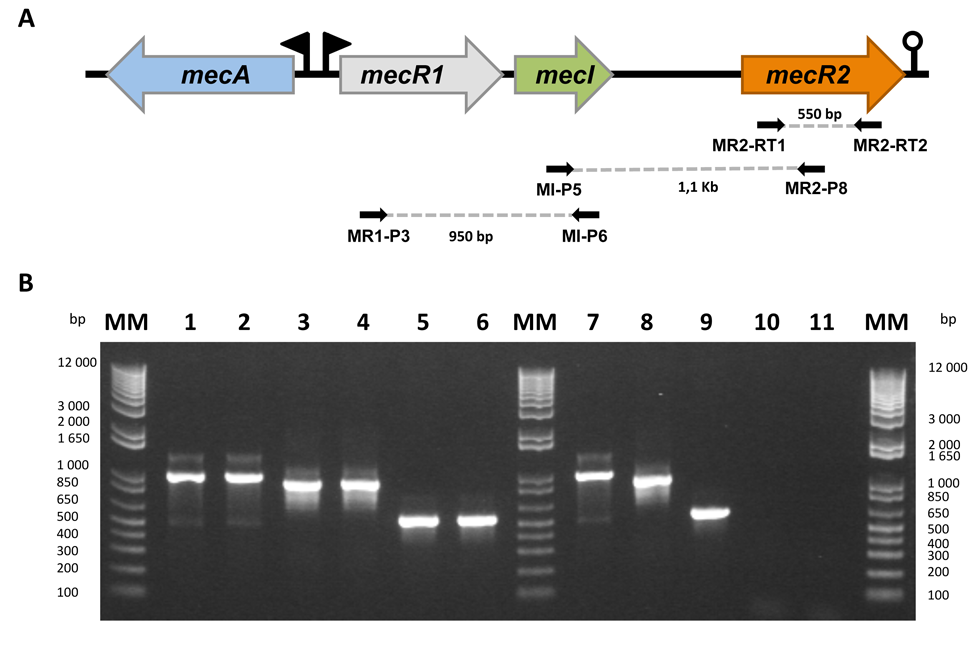

Supplement: Figure S2 — Transcriptional analysis of mecR2 . (A) Genetic organization of the mecA regulatory locus and location of primers used in the RT-PCR assays. (B) Gel electrophoresis of the RT-PCR products obtained with total RNA from strains N315 and HU25, respectively. MM, molecular weight marker (1 Kb DNA ladder); lanes 1–2, mecI-mecR2 co-transcript; lanes 3–4, mecR1-mecI co-transcript; lanes 5–6, mecR2 transcript; lanes 7–9, positive controls, PCR reactions using the same primer pairs and chromosomal DNA from strain N315 as template; lanes 10–11, negative control, RT-PCR reactions without the reverse-transcription step for total RNA preparations of strains N315 and HU25, respectively, using the primer pair MR2-RT1/MR2-RT2 (i.e. the one originating the smallest amplicon). (TIF) [file ppat.1002816.s002.tif]

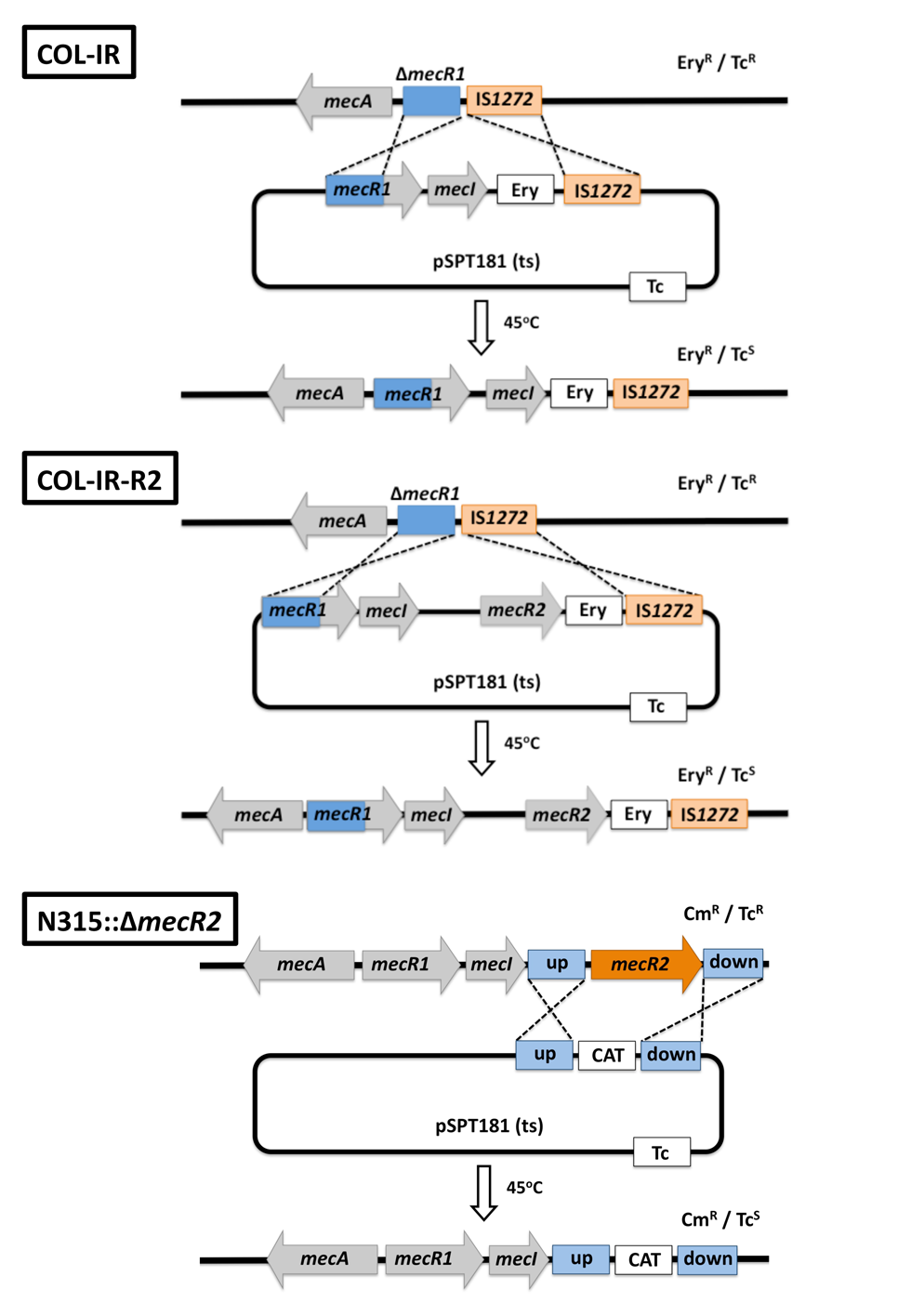

Supplement: Figure S3 — Insertion-deletion strategies used for the reconstruction of the mecA regulatory locus in the chromosome of strain COL and deletion of mecR2 from the chromosome of strain N315. Integration through homologous recombination of recombinant thermosensible plasmids was promoted at a non-permissive temperature (45°C) and with selection for tetracycline resistance (Tcr). Resolution of co-integrates was promoted at a permissive temperature (30°C) with selection for tetracycline resistance and segregation of the excised plasmids was promoted at 45°C without antibiotic selection. Colonies susceptible to tetracycline (Tcs) and resistant to erythromycin (Eryr) or chloramphenicol (Cmr) were selected for further analysis. (TIF) [file ppat.1002816.s003.tif]

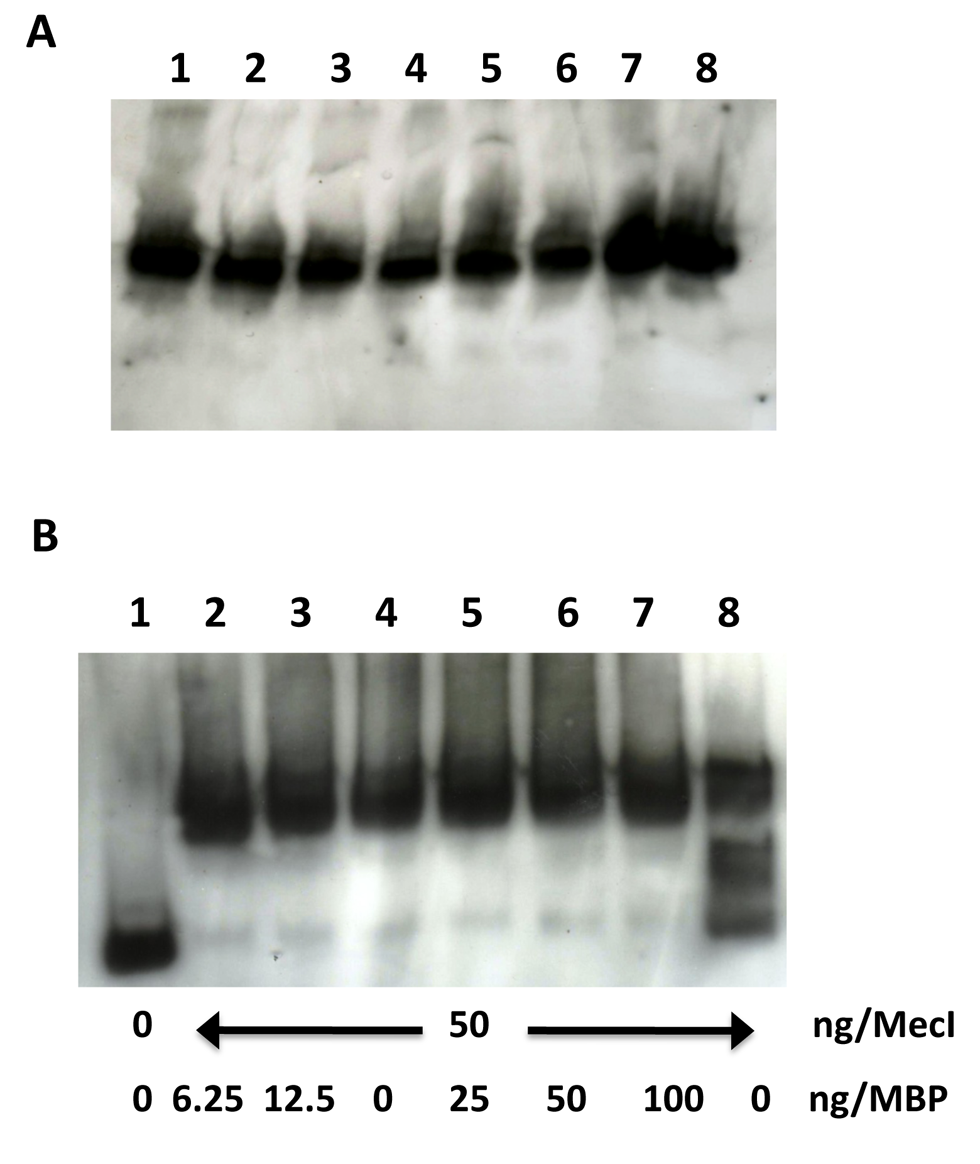

Supplement: Figure S4 — Control experiments for the electrophoretic mobility shift assays. (A) Binding of purified MecR2 to mecA promoter. Lane 1, negative control, labeled DNA only; lane 2, 0.001 µg of MecR2; lane 3, 0.01 µg of MecR2; lane 4, 0.05 µg of MecR2; lane 5, 0.1 µg of MecR2; lane 6, 0.25 µg of MecR2; lane 7, 0.5 µg of MecR2; lane 8, 1 ug of MecR2. (B) Binding of purified MecI to the labeled mecA promoter DNA sequence in the presence of MBP (Maltose-binding protein) at several molar ratios. MecI concentration was constant in all binding reactions (0.05 µg). Lane 1, negative control, labeled DNA only; lane 2, 8-fold excess of MecI; lane 3, 4-fold excess of MecI; lane 4, binding control, MecI only; lane 5, 2-fold excess of MecI; lane 6, equimolar amounts of MecI and MBP; lane 7, 2-fold excess of MBP; lane 8, control for specific binding, MecI with a 125 molar excess of unlabelled DNA. (TIF) [file ppat.1002816.s004.tif]
