# Supplementary material for: The Anti-Repressor MecR2 Promotes the Proteolysis of the mecA Repressor and Enables Optimal Expression of β-lactam Resistance in MRSA
Source: PLoS Pathog. 2012 Jul 26;8(7):e1002816. doi: 10.1371/journal.ppat.1002816 (PMC3406092; doi:10.1371/journal.ppat.1002816)
Supplement: Table S1 — Strains used in this study. (DOC) [file ppat.1002816.s005.doc]

**Table S1 – Strains used in this study**

| **Strain** | **Relevant characteristics** | **Source / Reference** |
| --- | --- | --- |
|  |  |  |
|  |  |  |
| *E. coli* DH5α | Recipient strain for recombinant plasmids | Stratagene |
| *E. coli* Bl21 (DE3) | Recipient strain for expression vector pCri8a | Novagene |
|  |  |  |
| *S. aureus* RN4220 | Restriction-deficient derivative of reference strain NCTC8325-4 | R. Novick |
| *S. aureus* COL | Prototype MRSA strain, homogeneous Oxa^r^, Δ*mecR*1 (no C-terminal inducer domain, ΨIS*1272* insertion), *mecI* negative, *mecR2* negative, β-lactamase negative, clone ST250-I | A. Tomasz [[1](#_ENREF_1)] |
| *S. aureus* N315 | Prototype MRSA strain, heterogeneous Oxa^r^, wild-type *mecR1-mecI*, *mecR2* positive, β-lactamase positive, clone ST5-II | K. Hiramatsu [[2](#_ENREF_2),[3](#_ENREF_3)] |
| *S. aureus* HU25 | Homogeneous Oxa^r^, wild-type *mecR1*, truncated *mecI*, *mecR2* positive, β-lactamase positive, clone ST239-III | [[3](#_ENREF_3),[4](#_ENREF_4)] |
| *S. aureus* USA100 | Epidemic MRSA strain NRS382, complete *mecR1-mecI* locus, *mecR2* positive, β-lactamase positive, clone ST5-II | NARSA.net [[5](#_ENREF_5)] |
| *S. aureus* USA200 | Epidemic MRSA strain NRS383, complete *mecR1-mecI* locus, *mecR2* positive, β-lactamase positive, clone ST36-II | NARSA.net [[5](#_ENREF_5)] |
| *S. aureus* USA600 | Epidemic MRSA strain NRS387, complete *mecR1-mecI* locus, *mecR2* positive, β-lactamase positive, clone ST45-II | NARSA.net [[5](#_ENREF_5)] |
| *S. aureus* HT0350 | Heterogeneous Oxa^r^, deleted *mecR1-mecI* (IS*431* insertion), β-lactamase negative, clone ST377-V | J. Étienne [[6](#_ENREF_6)] |
|  |  |  |
| COL  + pGC::*mecI* | COL overexpressing *mecI in trans* | [[3](#_ENREF_3)] |
| COL  + pGC::*mecI-mecR2* | COL co-overexpressing the *mecI*-*mecR2* locus *in trans* | This study |
| COL::*erm* | COL with *erm* gene inserted into the chromosome upstream to the *mecA* gene (control) | This study |
| COL::RI | COL with the *mecR1*-*mecI* locus inserted into the chromosome upstream to *mecA* gene | This study |
| COL::RI-R2 | COL with the *mecR1-mecI-mecR2* locus inserted into the chromosome upstream to *mecA* gene | This study |
| COL::RI  + pGC::*mecI* | COL::RI overexpressing *mecI in trans* | This study |
| COL::RI-R2  + pGC::*mecI* | COL::RI-R2 overexpressing *mecI in trans* | This study |

**Table S1 – cont.**

| **Strain** | **Relevant characteristics** | **Source / Reference** |
| --- | --- | --- |
|  |  |  |
| N315::Δ*mecR2* mut | N315 *mecR2* null mutant intermediate, β-lactamase negative | This study |
| N315::Δ*mecR2* | N315 *mecR2* deletion backcross, β-lactamase positive | This study |
| N315::Δ*mecR2*  + pSPT::*spac* | N315::Δ*mecR2* transformed with pSPT181 containing the P*spac* inducible promoter (control) | This study |
| N315::Δ*mecR2*  + pSPT::*mecR2* | N315::Δ*mecR2* overexpressing *mecR2 in trans* | This study |
| N315::Δ*mecR2*  + pSPT::*mecI-mecR2* | N315::Δ*mecR2* overexpressing *mecI* and *mecR2 in trans* | This study |
| N315::Δ*mecR2*  + pSPT::*spac*-*mecR2* | N315::Δ*mecR2* expressing *mecR2 in trans* from the inducible *Pspac* promoter | This study |
| COL::RI  + pSPT::*spac*-*mecR2* | COL::RI expressing *mecR2 in trans* from the inducible *Pspac* promoter | This study |
| USA100::Δ*mecR2* | USA100 *mecR2* null mutant, β-lactamase positive | This study |
| USA100::Δ*mecR2*  + pSPT::*spac*-*mecR2* | USA100::Δ*mecR2* expressing *mecR2 in trans* from the inducible *Pspac* promoter | This study |
| USA200::Δ*mecR2* | USA200 *mecR2* null mutant, β-lactamase positive | This study |
| USA200::Δ*mecR2*  + pSPT::*spac*-*mecR2* | USA200::Δ*mecR2* expressing *mecR2 in trans* from the inducible *Pspac* promoter | This study |
| USA600::Δ*mecR2* | USA600 *mecR2* null mutant, β-lactamase positive | This study |
| USA600::Δ*mecR2*  + pSPT::*spac*-*mecR2* | USA600::Δ*mecR2* expressing *mecR2 in trans* from the inducible *Pspac* promoter | This study |
| HT0350  + pSPT::*mecI* | HT0350 overexpressing *mecI in trans* | This study |
| HT0350  + pSPT::*mecI-mecR2* | HT0350 co-overexpressing *mecI* and *mecR2 in trans* | This study |
| HU25::Δ*mecR2* | HU25 *mecR2* null mutant, β-lactamase positive | This study |
| DH5α + pProEX::*mecI* | *E. coli* DH5α overexpressing *mecI* | This study |
| BL21 + pCri8a::*mecR2* | *E. coli* BL21 (DE3) overexpressing *mecR2* | This study |

**References**

1. de Lencastre H, Wu SW, Pinho MG, Ludovice AM, Filipe S, et al. (1999) Antibiotic resistance as a stress response: complete sequencing of a large number of chromosomal loci in *Staphylococcus aureus* strain COL that impact on the expression of resistance to methicillin. Microb Drug Resist 5: 163-175.

2. Kuwahara-Arai K, Kondo N, Hori S, Tateda-Suzuki E, Hiramatsu K (1996) Suppression of methicillin resistance in a *mecA*-containing pre-methicillin-resistant *Staphylococcus aureus* strain is caused by the *mecI*-mediated repression of PBP 2' production. Antimicrob Agents Chemother 40: 2680-2685.

3. Oliveira DC, de Lencastre H (2011) Methicillin-resistance in *Staphylococcus aureus* is not affected by the overexpression in trans of the *mecA* gene repressor: a surprising observation. PLoS One 6: e23287.

4. Teixeira LA, Resende CA, Ormonde LR, Rosenbaum R, Figueiredo AM, et al. (1995) Geographic spread of epidemic multiresistant *Staphylococcus aureus* clone in Brazil. J Clin Microbiol 33: 2400-2404.

5. McDougal LK, Steward CD, Killgore GE, Chaitram JM, McAllister SK, et al. (2003) Pulsed-field gel electrophoresis typing of oxacillin-resistant *Staphylococcus aureus* isolates from the United States: establishing a national database. J Clin Microbiol 41: 5113-5120.

6. Tristan A, Bes M, Meugnier H, Lina G, Bozdogan B, et al. (2007) Global distribution of panton-valentine leukocidin-positive methicillin-resistant *Staphylococcus aureus*, 2006. Emerg Infect Dis 13: 594-600.
