# Supplementary material for: The Anti-Repressor MecR2 Promotes the Proteolysis of the mecA Repressor and Enables Optimal Expression of β-lactam Resistance in MRSA
Source: PLoS Pathog. 2012 Jul 26;8(7):e1002816. doi: 10.1371/journal.ppat.1002816 (PMC3406092; doi:10.1371/journal.ppat.1002816)
Supplement: Table S2 — Plasmids used in this study. (DOC) [file ppat.1002816.s006.doc]

**Table S2 – Plasmids used in this study**

| **Plasmids** | **Relevant characteristics** | **Source** |
| --- | --- | --- |
| pGC-2 | *E. coli - S.aureus* shuttle vector, high-copy number, insert expression driven by bacteriophage promoters SP6 and T7, Ap^r^, Cm^r^ | P. Matthews |
| pSPT181 (ts) | *E. coli* - *S. aureus* shuttle vector, thermosensible, insert expression driven by bacteriophage promoter SP6, Ap^r^, Tc^r^ | [[1](#_ENREF_1)] |
| pSP64E | *E. coli* pSP64 vector with a 1.2 kb *Bam*H1-*Sal*I fragment containing the *erm* gene from Tn551 (integrative vector in *S. aureus*), Ap^r^, Ery^r^ | [[2](#_ENREF_2)] |
| pDH88 | *E. coli - B. subtilis* shuttle vector containing the IPTG inducible P*spac* promoter and the transcriptional repressor LacI, Ap^r^, Cm^r^ | [[3](#_ENREF_3)] |
| pSPT::*spac* | pSPT181 with 1.6 kb EcoR1-BamH1 fragment containing the IPTG inducible P*spac* promoter and the transcriptional repressor LacI from pDH88, Ap^r^, Tc^r^ | This study |
| pCri8a | pET30 (Invitrogen) derivative containing His6-GST-Tev fragment, Kan^r^ | [[4](#_ENREF_4)] |
| pGC::*mecI* | pGC2 with *mecI* gene from strain N315 | [[5](#_ENREF_5)] |
| pGC::*mecI-mecR2* | pGC2 with mecI gene and the *mecR2* locus from strain N315 | This study |
| pSPT::IS-*erm* | pSPT181 with a 0.6 kb fragment of IS*1272* and a 1.2 kb *Bam*H1-*Sal*I fragment containing the *erm* gene from pSP64E | This study |
| pSPT::IS-*erm*-Δ*mecR1* | pSPT::IS-*erm* with a 0.5 kb fragment of the N-terminal domain of *mecR1* | This study |
| pSPT::IS-*erm-mecI*-*mecR1* | pSPT::IS-*erm* with a 1.9 kb fragment containing *mecI*-*mecR1* from strain N315 | This study |
| pSPT::IS-*erm*-*mecR2*-*mecI-mecR1* | pSPT::IS-*erm* with a 3.5 kb fragment containing *mecR2*-*mecI*-*mecR1* from strain N315 | This study |
| pSPT::*cat*-Δ*mecR2* | pSPT181 vector containing the chloramphenicol acetyl transferase (Cm^r^) from pGC-2 flanked by 1.0 kb upstream and downstream vicinities of *mecR2* | This study |
| pSPT::*mecR2* | pSPT181 vector containing the *mecR2* gene from strain N315 | This study |
| pSPT::*mecI* | pSPT181 vector containing the *mecI* gene from strain N315 | This study |
| pSPT::*mecI-mecR2* | pSPT181 vector containing the *mecI* and *mecR2* genes from strain N315 | This study |
| pSPT::*spac*-*mecR2* | pSPT181 vector containing the *mecR2* gene from strain N315 under control of the P*spac*  inducible promoter | This study |
| pProEX::*mecI* | Expression vector pP_RO_EX^TM^ Hta (Invitrogen) with His_6_ tag N-terminal fusion to *mecI* gene from strain N315, Ap^r^ | [[6](#_ENREF_6)] |
| pCri8a::*mecR2* | pCri8a with *mecR2* gene from strain HU25 | This study |

**References**

1. Janzon L, Arvidson S (1990) The role of the delta-lysin gene (*hld*) in the regulation of virulence genes by the accessory gene regulator (*agr*) in *Staphylococcus aureus*. EMBO J 9: 1391-1399.

2. Pinho MG, de Lencastre H, Tomasz A (2000) Cloning, characterization, and inactivation of the gene *pbpC*, encoding penicillin-binding protein 3 of *Staphylococcus aureus*. Journal of Bacteriology 182: 1074-1079.

3. Henner DJ (1990) Inducible expression of regulatory genes in *Bacillus subtilis*. Methods Enzymol 185: 223-228.

4. Kapust RB, Waugh DS (1999) *Escherichia coli* maltose-binding protein is uncommonly effective at promoting the solubility of polypeptides to which it is fused. Protein Science 8: 1668-1674.

5. Oliveira DC, de Lencastre H (2011) Methicillin-resistance in *Staphylococcus aureus* is not affected by the overexpression in trans of the *mecA* gene repressor: a surprising observation. PLoS One 6: e23287.

6. García-Castellanos R, Marrero A, Mallorquí-Fernández G, Potempa J, Coll M, et al. (2003) Three-dimensional structure of MecI. Molecular basis for transcriptional regulation of staphylococcal methicillin resistance. J Biol Chem 278: 39897-39905.
