# Supplementary material for: The Anti-Repressor MecR2 Promotes the Proteolysis of the mecA Repressor and Enables Optimal Expression of β-lactam Resistance in MRSA
Source: PLoS Pathog. 2012 Jul 26;8(7):e1002816. doi: 10.1371/journal.ppat.1002816 (PMC3406092; doi:10.1371/journal.ppat.1002816)
Supplement: Table S3 — Primers used in this study. (DOC) [file ppat.1002816.s007.doc]

**Table S3 – Primers used in this study**

| **Name** | **Sequence (5’ → 3’) *** |
| --- | --- |
| MA-P1 | AAATCGATGTAAAGGTTGGC |
| MA-P2 | GTTCTG CAG TAC CGGATT TG |
| MA-RT1 | AACATTGATCGCAACGTTCAAT |
| MA-RT2 | TGGTCTTTCTGCATTCCTGGA |
| MA-PF1 | ATA TCG TGA GCA ATG AAC TG |
| MA-PR1 | TAT ATA CCA AAC CCG ACA AC |
|  |  |
| MI-P1 | TATAGAATTCGCACAACAAATTTCTGAGCG |
| MI-P2 | GATCGGATCCATGCATATGGATTTCACTGG |
| MI-P3 | TCTAGGATCCTCAACGACTTGATTGTTTC |
| MI-P4 | TAATCTGCAGCACAACAATTTTCTCAG |
| MI-P5 | GCGGTTTCAATTCACTTGTC |
| MI-P6 | TGGtttttggactccagtcc |
| MI-BTH1 | TATATCTAGAGGATAATAAAACGTATGAAATATCATCTGC |
| MI-BTH2 | TCTAGGTACCCGTTTATTCAATATATTTCTCAATTCTTCTATTTCATC |
| MI-Box1 | TTGACATAAATACTACATTTGTAATATACTACAAATGTA |
| MI-Box2 | AGACTACATTTGTAG TATATTACAAATGTAGTATTT ATG |
| MR-P1 | TATACCCGGGAAAGTTCGTCATTGGAATCG |
| MR-P2 | GATCGGATCCATACGCTTGTTTCGATTAGG |
| MR-P3 | GCACTTTATGATTCAATGCC |
|  |  |
| MR2-P1 | GTTAGGATCCGCTATCAACATTTACCAGCA |
| MR2-P2 | TATAGTCGACCAAAATACTAGAAATCGTTGCC |
| MR2-P3 | TAATCTGCAGCACAACAATTTTCTCAG |
| MR2-P4 | TATAGGATCCTGCTGGTAAATGTTGATAGC |
| MR2-P5 | TAATCTCGAGTTAGAAGTCTTACACACTCC |
| MR2-P6 | ATTACCCGGGCTATCAACATTTACCAGCA |
| MR2-P7 | TATACCCGGGTATGGGGTAGGCAATTATGG |
| MR2-P8 | CTACTAACCTTTTCATCAGG |
| MR2-RT1 | AATGAAGCGAATCTTTCAGC |
| MR2-RT2 | AATTGCTAATGTACCACCTAGC |
| MR2-RT3 | ccattccatgaaactgaagg |
| MR2-RT4 | aacgctgaaagattcgcttc |
| MR2-BTH1 | TCTATCTAGAGTACAATTATTTTGATGGTAATGTC |
| MR2-BTH2 | TCTAGGTACCCGTGCTTTTATATCTAAGTAAATATCATTAATCTGTAG |
| MR2-Cri1 | TTAATACGCCATGGTTGATAAAAAAGAGTGCTAAGG |
| MR2-Cri2 | GGATCCTCGAGCTATTATGCTTTTATATCTAAG |
|  |  |
| IS1272-P1 | TATACTGCAGATGATTGTTCAGAATGTCC |
| IS1272-P2 | TATAGTCGACAAGAGTTAAGAGCCATTGC |
|  |  |
| CAT-P1 | TAATCTGCAGAAGAAAGCAGACAAGTAAGC |
| CAT-P2 | TGTAGTCGACAAACCTTCTTCAACTAACGG |
|  |  |
| pta-RT1 | AGAAGCAATCATTGATGGCGA |
| pta-RT2 | ACCTGGCGCTTTTTTCTCAG |

* Restriction sites are underlined. For primers MI-Box1/MI-Box2 the MecI protected sequences are underlined.
