# Supplementary material for: The Anti-Repressor MecR2 Promotes the Proteolysis of the mecA Repressor and Enables Optimal Expression of β-lactam Resistance in MRSA
Source: PLoS Pathog. 2012 Jul 26;8(7):e1002816. doi: 10.1371/journal.ppat.1002816 (PMC3406092; doi:10.1371/journal.ppat.1002816)
Supplement: Table S4 — Strains and plasmids used in the bacterial two-hybrid assays. (DOC) [file ppat.1002816.s008.doc]

**Table S4 - Strains and plasmids used in the bacterial two-hybrid assays**

| **Strains / Plasmids** | **Relevant characteristics** | **Source** |
| --- | --- | --- |
| E. coli BTH 101 | Reporter strain, cya^-^ | [[1](#_ENREF_1)] |
| pUT18 | BTH plasmid; N-terminal *cyaA*-T18 fusion; Amp^r^ | [[1](#_ENREF_1)] |
| pUT18c | BTH plasmid; C-terminal *cyaA*-T18 fusion; Amp^r^ | [[1](#_ENREF_1)] |
| pKT25 | BTH plasmid; C-terminal *cyaA*-T25 fusion; Kan^r^ | [[1](#_ENREF_1)] |
| pKNT25 | BTH plasmid; N-terminal *cyaA*-T25 fusion; Kan^r^ | [[1](#_ENREF_1)] |
| p18Zip | BTH control plasmid; Amp^r^ | [[1](#_ENREF_1)] |
| p25Zip | BTH control plasmid; Kan^r^ | [[1](#_ENREF_1)] |
| pUT18::mecI | BTH plasmid containing *mecI*::*cyaA*-T18 fusion | This study |
| pUT18c::mecI | BTH plasmid containing *cyaA*-T18::*mecI* fusion | This study |
| pKT25::mecI | BTH plasmid containing *cyaA*-T25::*mecI* fusion | This study |
| pKNT25::mecI | BTH plasmid containing *mecI*::cyaA-T25 fusion | This study |
| pUT18::mecR2 | BTH plasmid containing *mecR2*::cyaA-T18 fusion | This study |
| pUT18c::mecR2 | BTH plasmid containing cyaA-T18::*mecR2* fusion | This study |
| pKT25::mecR2 | BTH plasmid containing cyaA-T25::*mecR2* fusion | This study |
| pKNT25::mecR2 | BTH plasmid containing *mecR2*::cyaA-T25 fusion | This study |

1. Karimova G, Pidoux J, Ullmann A, Ladant D (1998) A bacterial two-hybrid system based on a reconstituted signal transduction pathway. Proc Natl Acad Sci U S A 95: 5752-5756.
